# A multibranch and multiscale neural network based on semantic perception for multimodal medical image fusion

**DOI:** 10.1038/s41598-024-68183-3

**Published:** 2024-07-30

**Authors:** Cong Lin, Yinjie Chen, Siling Feng, Mengxing Huang

**Affiliations:** School of Information and Communication Engineering, Hainan University, Haikou, 570228 Hainan China

**Keywords:** Multimodal medical image, Image fusion, High-level vision task, Multibranch features, Semantic aware, Bioluminescence imaging, Image processing

## Abstract

Medical imaging is indispensable for accurate diagnosis and effective treatment, with modalities like MRI and CT providing diverse yet complementary information. Traditional image fusion methods, while essential in consolidating information from multiple modalities, often suffer from poor image quality and loss of crucial details due to inadequate handling of semantic information and limited feature extraction capabilities. This paper introduces a novel medical image fusion technique leveraging unsupervised image segmentation to enhance the semantic understanding of the fusion process. The proposed method, named DUSMIF, employs a multi-branch, multi-scale deep learning architecture that integrates advanced attention mechanisms to refine the feature extraction and fusion processes. An innovative approach that utilizes unsupervised image segmentation to extract semantic information is introduced, which is then integrated into the fusion process. This not only enhances the semantic relevance of the fused images but also improves the overall fusion quality. The paper proposes a sophisticated network structure that extracts and fuses features at multiple scales and across multiple branches. This structure is designed to capture a comprehensive range of image details and contextual information, significantly improving the fusion outcomes. Multiple attention mechanisms are incorporated to selectively emphasize important features and integrate them effectively across different modalities and scales. This approach ensures that the fused images maintain high quality and detail fidelity. A joint loss function combining content loss, structural similarity loss, and semantic loss is formulated. This function not only guides the network in preserving image brightness and texture but also ensures that the fused image closely resembles the source images in both content and structure. The proposed method demonstrates superior performance over existing fusion techniques in objective assessments and subjective evaluations, confirming its effectiveness in enhancing the diagnostic utility of fused medical images.

## Introduction

Medical imaging is a crucial tool in modern medical diagnosis and treatment. With the advancement of medical imaging technologies, various medical images with distinct features emerge, generated by different principles and devices. Different modalities of medical images possess unique characteristics and contain specific information. Common medical imaging techniques include Magnetic Resonance Imaging (MRI), Computed Tomography (CT) and Positron Emission Computed Tomography (PET). CT images provide transparent anatomical relationships and excel at imaging dense structures like bones and calcifications, while MRI images offer excellent soft tissue resolution for imaging soft tissues. However, a single modality image often falls short of conveying sufficient information. To make accurate diagnoses, doctors frequently must repeatedly observe and compare medical images of the same area from different modalities. This process increases their workload and reduces the efficiency of consultations. Multimodal medical image fusion technology addresses this by combining images from two or more different modalities of the same object. The fused image retains information from different modality source images as much as possible, better-highlighting organs or lesion areas within the image. The technology is vital in facilitating advanced visual tasks in medical imaging, such as segmentation, classification, and detection, thereby enhancing the efficiency of subsequent medical image analysis^1^.

In the early stages of research on medical image fusion, the focus was primarily on spatial domain-based methods. These methods apply fusion rules directly to the pixels of source images, where the pixel values of source images are combined to produce the fused image. Simple maximum and minimum methods, weighted averaging, Independent Component Analysis (ICA), Principal Component Analysis (PCA) and fuzzy logic, among others, are examples of spatial domain-based medical image fusion methods^2–4^. These methods operate directly on pixel values, offering low complexity and fast fusion capabilities. However, the resulting images often have low signal-to-noise ratios and suffer from spectral distortion and spatial distortion. Researchers shifted their focus to transform domain-based methods to address the spectral and spatial distortions in spatial domain-based fusion images. Transform domain methods involve applying specific transformations to input images to obtain low-frequency and high-frequency coefficients. These coefficients are then fused, and the fused results are inverse-transformed to generate the final fused image. Transformation methods used for input images include wavelet transform, contourlet transform, shearlet transform, and more^5–7^. Each of these transformations has its drawbacks, leading to improvements and extensions. For example, the stationary wavelet transform was used to address the lack of shift-invariance in wavelet transforms when representing directional features. The linear phase properties of biorthogonal wavelets were utilized to decompose and reconstruct images^8,9^. Bhateja et al.^10^ proposed that wavelet and ridgelet transforms have complementary capabilities in extracting edge features, and Mathiyalagan^11^ introduced an image fusion algorithm using wavelet and ridgelet transforms to transform the low and high-frequency subbands of images separately. To mitigate information loss caused by transformations, a series of non-subsampled transform methods, such as Non-Subsampled Wavelet Transform (NSWT), Non-Subsampled Contourlet Transform (NSCT), and Non-Subsampled Shearlet Transform (NSST), were applied in the transformation process of image fusion algorithms^12–14^. Later approaches based on pyramids and multi-scale decomposition further improved the utilization of image information by increasing the number of scales in transformations, but they could introduce artefacts or amplify image noise. The methods mentioned above can be classified as traditional medical image algorithms, and they share a common drawback: lack of generalization. Researchers need to design fusion rules tailored to specific scenarios manually, and using the same rules for different scenarios might result in suboptimal fusion outcomes.

In recent years, with the advancement of convolutional neural network (CNN) and deep learning technology, these innovations have demonstrated remarkable performance in computer vision and image processing. They have also assumed a pivotal role in medical image fusion. Non-end-to-end deep learning-based methods for medical image fusion leverage the feature extraction capabilities of deep neural networks. These methods involve the utilization of neural networks or the amalgamation of neural networks with image transformation techniques to extract image features from source images^15–17^. Following the extraction of features through manually designed fusion rules, the fused features are then used to reconstruct the fused image. However, the features extracted by neural networks within this category of methods do not guarantee optimality and have not overcome the limitations of poor generalization inherent in traditional methods. End-to-end deep learning-based approaches for medical image fusion have emerged in response to this challenge. An end-to-end image fusion approach implies that the network takes source images as inputs and produces fused images as outputs. Deep learning models within this framework learn the mapping from source images to fused images, thereby bypassing the constraints posed by manually designed fusion rules. Such approaches can be categorized into those based on CNN and those based on generative adversarial networks. CNN-based medical image fusion methods implicitly conduct feature extraction, feature fusion, and image fusion through convolutional neural networks. Through the backpropagation of loss functions, these methods iteratively update network weight parameters to progressively align the fused image with the desired outcome^18–20^. Conversely, generative adversarial network-based medical image fusion methods engage in an adversarial game between source and fused images. A discriminator compels the generator to produce fused results consistent with the target probability distribution. This process implicitly achieves the extraction, fusion, and image reconstruction of source image features. These advancements signify substantial progress in the field of medical image fusion, harnessing the capabilities of deep learning to more effectively and efficiently manage feature extraction, fusion, and image reconstruction tasks^21–23^.

In order to enhance the quality of fused images by extracting a broader range of image features, researchers have undertaken investigations from various perspectives. The process of feature extraction inevitably leads to information loss^24^. Extracting and fusing multi-scale hierarchical features can effectively alleviate this problem. By inputting the source image into a convolutional network and hierarchically extracting source image features, followed by fusing features at the same hierarchical level, a multi-scale feature representation of the fused image can be obtained^25^. Another approach to enhancing network feature extraction capabilities involves improving the network architecture. Xia et al.^26^ proposed a medical image fusion method using a deeply stacked CNN. This method avoids the loss of feature information caused by downsampling layers in the CNN. Fu et al.^27^ introduced a multi-scale residual pyramid attention network for medical image fusion. Residual networks, through skip connections, can mitigate the problem of gradient vanishing in deep networks and enhance the expressive capacity of the network. Tang et al.^28^ pointed out that convolutional neural networks have a limited ability to retain global contextual information. Therefore, they incorporated Transformers to enhance the ability of the network to extract global information. They also modified conventional convolutions to adaptive convolutions, further modelling the long-range dependency relationship in multi-modal information. In medical image fusion methods based on generative adversarial networks (GANs), research has primarily focused on addressing the issue of GANs fusing only partial information from source images and enhancing information in fused images. Source images of different modalities possess distinct feature information. The competition between a single generator and discriminator could result in fused images resembling a single image. To tackle this, Ma et al.^21^ proposed a fusion method employing a conditional GAN with dual discriminators. This approach treats image fusion as a specific adversarial process involving two networks (one generator and two discriminators) based on conditional GANs. Li et al.^22^ designed two sets of generators and discriminators. The first generator generates structurally informed images based on pre-fused images, while the first discriminator measures the relative displacement between generated images and the first modality images. The second generator generates images with enhanced gradient information based on pre-fused images, and the second discriminator measures the displacement of generated images relative to the second modality images. Huang et al.^23^ introduced MGMDcGAN, where two sets of conditional GANs focus on structural and functional information of the source images, respectively.

An evolving and refined feature extraction network can extract richer texture and detail information from images. However, during the fusion process, feature information from two different modal images inevitably conflicts to some extent, necessitating the omission of certain information. Existing methods train networks based on predefined loss functions, lacking a profound understanding of image content, and thus cannot ensure that the retained information is relevant to the fused image. Moreover, whether based on convolutional neural networks or generative adversarial networks, these methods are limited to extracting intuitive image information, neglecting extracting higher-level semantic information. Advanced semantic information in images refers to the meaning and content expressed by the image, involving the comprehension of objects in the image, as demonstrated by visual tasks like object detection and segmentation. In various image processing tasks, introducing high-level semantic information has positively impacted image processing, enhancing the quality of resultant images. Driven by the concept of high-level visual tasks guiding low-level image processing, Tang et al.^29^ proposed a semantic-aware fusion framework for infrared and visible light images. However, this method has stringent dataset requirements and does not apply to medical image fusion. To date, no research has harnessed image semantic information in medical image fusion. Hence, this paper introduces a CT-MRI medical image fusion method based on multi-branch, multi-scale feature extraction and semantic information. The approach leverages image segmentation tasks to acquire semantic information from source images and employs an improved multi-branch, multi-scale feature extraction network to extract features from source images. A feature fusion and reconstruction network is devised to correspond to the extracted multi-branch and multi-scale features. Furthermore, a joint loss function is designed, where semantic information acquired from image segmentation tasks contributes as part of the loss function, transmitting to the image fusion network. Experimental results demonstrate the excellence of the proposed method in objective metrics and visual effects, showcasing superior comprehensive performance compared to existing medical image fusion methods.

Compared to existing medical image fusion methods, the main contributions of this paper can be summarized as follows:

- An approach that leverages unsupervised image segmentation is introduced to acquire semantic information from source images in this paper. The semantic information is then integrated into the medical image fusion network, assisting in the training process. This integration improves the ability of the network to generate better-fused images.
- The paper proposes a medical image fusion network based on a multi-branch, multi-scale structure. This design enhances feature extraction by utilizing multiple branches for feature extraction, leading to the extraction of richer image feature information. The corresponding feature fusion and reconstruction network uses a multi-attention mechanism to select and process practical features from the extracted image features.
- A joint loss function comprising content loss, structural similarity loss and semantic loss is designed. The content loss ensures appropriate brightness in the fused image and encourages the inclusion of richer details and textures. The structural similarity loss complements the content loss by making the fusion result resemble the two source images as much as possible. The semantic loss function incorporates semantic information from image segmentation tasks, further enhancing the quality of the fused image.

The remaining chapters of the thesis are arranged as follows. In the second section, medical image fusion and related work are sorted out and introduced. “Methodology” section gives a detailed introduction to the proposed method. The fusion effect of the proposed method is shown in “Experiments” section, and the proposed method is compared with advanced medical image fusion methods on different indicators. The results of ablation experiments are also shown to demonstrate the effectiveness of the method. The summary of this paper is given in “Conclusion” section.

## Related work

### Task-driven and semantic-aware image processing methods

Applying task-driven approaches and semantic perception has yielded successful results in low-level image processing tasks such as dehazing, denoising, and super-resolution. For instance, Zhang et al.^30^ utilized semantic information as color constraints in dehazing, enabling the dehazing network to faithfully restore textures and eliminate unnatural appearances of specific objects within images. Lee et al.^31^ introduced a task-driven image enhancement network that is connected to higher-level vision tasks, enhancing the task-driven training strategy so that semantic information from advanced visual tasks can robustly guide task models for high-quality image restoration and precise perception. Ren et al.^32^ proposed a stacked generator network for image restoration, incorporating semantic information to improve issues of unnatural content in the resultant images. Liu et al.^33^ presented a task-driven image denoising method that employs a two-module cascade for image denoising and high-level visual tasks. This approach utilizes high-level visual information to guide the denoising network in producing visually appealing outcomes. Haris et al.^34^ adopted an object detection task to drive image super-resolution, performing joint optimization of image super-resolution and object detection. This approach enhances the effectiveness of image super-resolution and facilitates more accurate results in the object detection task for post-super-resolution images.

Furthermore, research efforts have also applied task-driven approaches and semantic perception to image fusion. Tang et al.^29^ proposed a semantic-aware framework for fusing infrared and visible light images. They utilized image segmentation to acquire semantic information, which was then employed to guide the image fusion process, effectively enhancing the performance of fused images in higher-level visual tasks. Building upon this foundation, Tang et al.^35^ integrated the semantic requirements of image registration, image fusion, and advanced visual tasks, introducing a novel method for image registration and fusion. Sun et al.^36^ introduced an object detection-driven network for fusing infrared and visible light images. This method cascaded the image fusion network with detection networks from both modalities, utilizing target-relevant semantic information learned within the object detection networks to guide multi-modal image fusion.

Integrating semantic information from advanced visual tasks with low-level image processing enables more effective utilization of semantics during image processing. This emphasis on image content and semantic structure enhances the semantic coherence of processing results, thereby improving processing effectiveness and accuracy. However, as far as we know, there has yet to be any research that has introduced task-driven approaches and semantic perception into the field of medical image fusion. Additionally, it provides comprehensive information about critical features like shape, size and spatial distribution because image segmentation detects the presence and location of objects and accurately outlines and delineates their contours and boundaries. This is valuable for doctors to understand targets holistically, supporting diagnostic and treatment decisions. Hence, semantic information from image segmentation tasks suits medical image fusion applications.

### Multi-branch and multi-scale feature extraction

Multi-scale feature extraction is commonly employed in computer vision and image processing. Features extracted at different scales provide information at various levels. Lower-scale features typically encompass local details and texture information, while higher-scale features focus more on global structures and abstract information. Multi-scale feature extraction combines these diverse scale-based pieces of information to create a more comprehensive and enriched feature representation. Multi-branch feature extraction incorporates multiple branches within a network, enabling inter-branch information exchange and collaborative learning. Mutual supplementation and correction occur by facilitating the transfer and exchange of information between branches, enhancing the overall feature expression capability. Meanwhile, multi-branch and multi-scale feature extraction combines multiple branches and multi-scale feature extraction techniques. Its objective is to simultaneously harness the benefits of multiple branches and various scales of feature information. This approach aims to achieve a more enriched and comprehensive feature representation, reinforcing an adaptability of the model to input data of varying scales and complexities. This, in turn, reduces noise and enhances the performance of the model in tasks related to computer vision and image processing.

The term of multi-branch and multi-scale feature extraction network encompasses a category of networks widely employed across various domains, with distinct network structures in each approach. For instance, Wang et al.^37^ devised a multi-branch and multi-scale pyramid network for pedestrian re-identification. This task demands deep features representing representative and robust pedestrians, encompassing global contour information and local details. Their method involves duplicating the main network structure to construct a multi-branch deep model and a lightweight multi-scale feature pyramid structure. Jia et al.^38^ proposed a multi-branch, multi-scale CNN for motion intention classification. This network effectively decodes raw electroencephalogram signals, addressing issues related to subject and temporal differences in a parallel processing manner, along with the representation of distinct frequency band information. Chen et al.^39^ involves a cascaded convolutional neural network that integrates U-net architecture with bidirectional attention guidance and refinement residual networks to achieve precise segmentation of breast ultrasound images. The method employs a unique structure to combine low-level and high-level features to enhance segmentation performance, effectively managing global and local feature dependencies. Jiang et al.^40^ introduced an object tracking framework based on a multi-branch, multi-scale twin CNN. This framework, rooted in relation mining, establishes a multi-branch tracking structure where branches mutually validate through their combinations. The multi-scale perception module aids the tracker in effectively handling scale variations. Ghaderizadeh et al.^41^ presented a multi-scale dual-branch residual spectral-spatial network for hyperspectral image classification. The dual-branch structure is employed to extract useful spectral-spatial features from hyperspectral images, while the multi-scale network is employed to capture multi-scale information, enhancing the accuracy of complex hyperspectral data classification. Similar to the work in this paper, Li et al.^42^ proposed a dual-branch multi-scale residual network for fusing anatomical and functional medical images. The two branches of the network are differentially designed, with one branch utilizing a multi-scale approach for feature extraction while the other employs only convolutions. Chen et al.^43^ uses a multi-branch encoding network and a refined decoding network to handle variable image quality and complex structural characteristics typical of ultrasound images. By using a multi-scale feature input pyramid and deep supervision modules, this method enhances the ability to generalize and effectively segment images under varying conditions, which is relevant for multi-scale and multi-branch feature extraction methods. It should be noted that while the method proposed in this paper also utilizes multi-branch and multi-scale feature extraction for medical image fusion, the network structures employed are entirely distinct.

### Attention mechanism

In deep learning, the attention mechanism refers to the tendency to focus on distinct parts when dealing with large volumes of information. It has been widely applied across various application domains. With the advancement of deep neural networks, numerous attention mechanisms emerge. Existing attention mechanisms can be categorized into types such as channel attention, spatial attention, and temporal attention. Hu et al.^44^ introduced a channel attention mechanism named SEnet, which stands for Squeeze-and-Excitation. Its core component is the SE block, which gathers global information, captures channel relationships and enhances representation capability. To augment informative channels and significant regions, Woo et al.^45^ proposed the Convolutional Block Attention Module (CBAM). This module combines channel attention and spatial attention in a stacked manner, decoupling channel attention maps and spatial attention maps to improve computational efficiency. It also leverages global pooling to incorporate spatial global information. In order to address limitations in attention confined to local relationships and perform poorly in capturing long-range dependencies, Hou et al.^46^ introduced Coordinating Attention. This approach embeds positional information into channel attention, enabling the network to focus on large, significant regions with minimal computational costs.

In the fields of computer vision and image processing, attention mechanisms have found widespread applications in tasks such as image denoising, image super-resolution, object detection, and image segmentation^47–50^. Chen et al.^51^ introduces a hybrid adaptive attention module which combines channel self-attention and spatial self-attention mechanisms. This dual attention strategy not only focuses on distinct channel features but also on spatial features, improving the segmentation accuracy significantly by adapting to complex image characteristics. Furthermore, within the realm of image fusion methods, attention mechanisms have been employed for the fusion of multimodal image feature information. Wang et al.^52^ proposed an image fusion network based on dual non-local attention dense residuals for infrared and visible light image fusion. The attention mechanism in their approach focuses on prominent infrared targets and distinct visible details, enhancing the feature maps obtained by the encoder network. Ma et al.^53^ introduced an image fusion framework based on cross-domain long-range learning and the Swin Transformer. They utilized self-attention and cross-attention mechanisms to achieve feature fusion within and across domains. To address the challenge of restoring texture details while correcting color distortion in multi-exposure images, Liu et al.^54^ proposed an attention-guided global-local adversarial learning network. This method employs hierarchical attention to guide the fusion of image features. In the approach presented in this paper, multiple attention mechanisms are employed to fuse image features from different modalities, branches and scales.

## Methodology

This section describes the proposed method for fusing multi-modal medical images, named DUSMIF. Firstly, an overview of the general framework of the method is presented. Subsequently, the structure of the fusion network and the details of each module are elaborated upon, and the segmentation network used is briefly introduced. Finally, an explanation of the loss functions is provided.

### Overall framework

**Figure 1:**
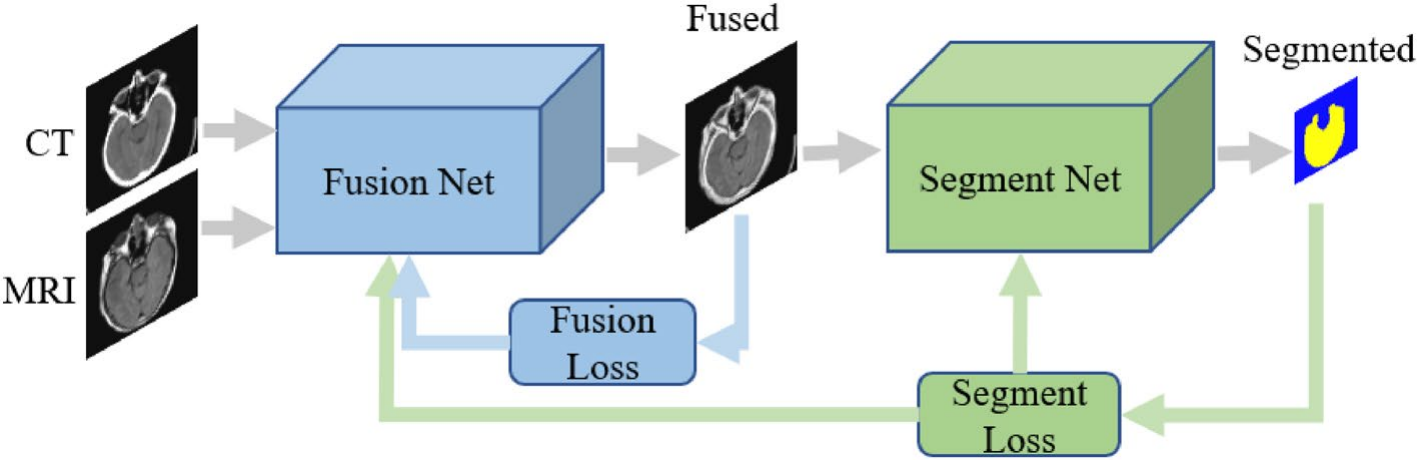
The overall framework of the proposed method.

The DUSMIF method proposed in this paper consists of a multi-scale multi-branch image fusion network and an image segmentation network. The image fusion network extracts and fuses features from multi-modal images to achieve reconstruction. It serves as the core and focus of this method. On the other hand, the image segmentation network is utilized to obtain semantic information from the fused image. As described in “Task-driven and semantic-aware image processing methods” section, the effectiveness of this approach is thoroughly validated by transferring semantic information to relevant image processing tasks through loss functions. Similarly, our method employs segmentation losses derived from the image segmentation network to feed semantic information to the fusion network. The overall framework of our method and the flow of information are illustrated in Fig. 1.

During the training of the network, CT and MRI source images are passed through the image fusion network to generate fused images, from which the fusion loss is computed. These fused images then undergo the image segmentation network to generate pixel-level segmentation labels and obtain segmentation loss. The segmentation loss, derived from the image segmentation task and containing semantic information about the images, is also referred to as semantic loss. The fusion loss and segmentation loss collectively form the loss function of the image fusion network, guiding the update of network parameters. Conversely, the loss function of the image segmentation network only comprises the segmentation mentioned above loss. It is important to note that the ultimate output of this method, after processing multi-modal medical images through the network, is the fused image. The segmentation labels associated with the fused image are intermediary products generated during training for acquiring semantic loss.

Due to the simultaneous utilization of the segmentation loss as the loss function for both networks, training the fusion and segmentation networks together can lead to an issue of clarity training objectives. This ambiguity arises from the inability to distinguish whether a reduction in segmentation loss is due to improvements in the fusion or segmentation networks. Consequently, the training outcomes might overly specialize the image segmentation network, negatively impacting the performance of the image fusion network. While employing a pre-trained image segmentation network can mitigate this problem, a fixed segmentation network might gradually become less adaptable to variations in input images, introducing certain biases in semantic results. To address this concern, the approach in this paper adopts an alternating training scheme during the network training process. Specifically, within the same epoch, only one of the two networks (either the image fusion network or the image segmentation network) is trained. After a predetermined number of epochs, the training focus alternates between them. This approach helps better balance the two networks and their respective training objectives.

### Multi-branch and multi-scale image fusion network

**Figure 2:**
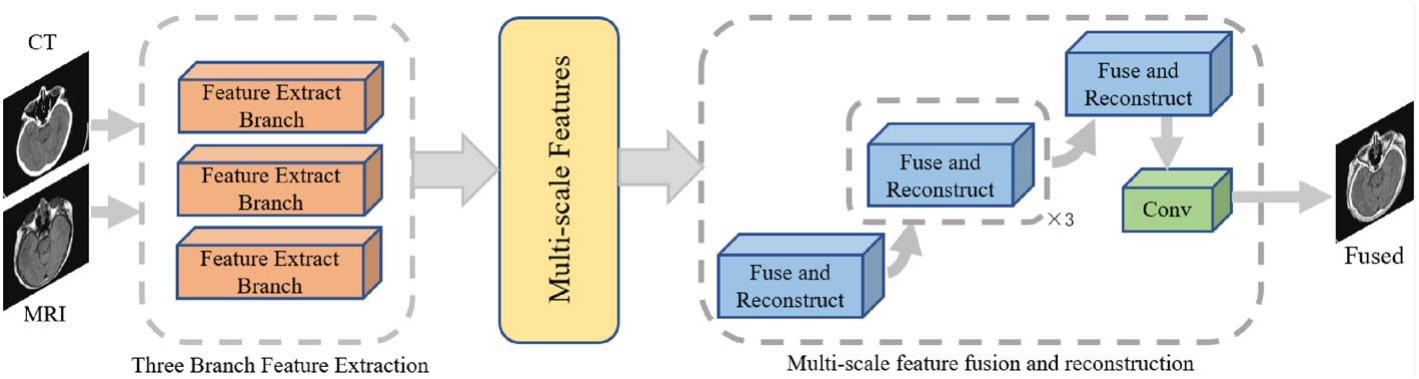
The structure of fusion network.

In Fig. 2, the multi-branch and multi-scale fusion network structure for CT and MRI medical images is presented. The fusion network comprises a multi-branch and multi-scale feature extraction network and a corresponding multi-branch and multi-scale feature fusion reconstruction network.

As explained in “Multi-branch and multi-scale feature extraction” section, employing multiple branches for feature extraction contributes to obtaining a more comprehensive and enriched feature representation. However, an increased number of branches implies higher computational demands, while the improvements gained diminish with the growing number of branches. Balancing resource consumption with the enhancement in feature extraction capability, the proposed method in this paper employs three feature extraction branches in the feature extraction network, designed with appropriate lightweight considerations. Each feature extraction branch processes the input images and outputs features at five scales. Through the multi-branch and multi-scale feature extraction network, the input multi-modal medical images yield six sets of image features across five scales, with each modality possessing three sets of features.

Corresponding to the extracted feature scales, the multi-branch and multi-scale image fusion reconstruction network comprises five fusion reconstruction blocks. In each fusion reconstruction block, multiple attention mechanisms are employed for inter-modality feature fusion, inter-branch feature fusion, and intra-scale feature fusion with the previous scale. These multiple attention mechanisms during fusion allow for the selective integration of extracted rich features and emphasize their crucial aspects. The convolutional block at the end of the fusion reconstruction network consists of three convolutional layers (each with a 3 $\times$ 3 kernel size), LeakyReLU activation functions and batch normalization. This block is responsible for constructing the mapping from fused features to the final fused image.

#### Feature extraction branch

**Figure 3:**
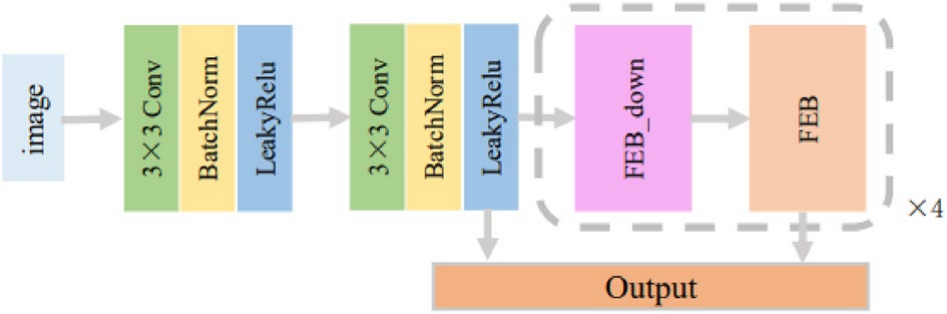
The structure of feature extraction branch.

The feature extraction branch proposed by this method consists of two convolutional layers and four feature extraction pairs. Each feature extraction pair consists of a downsampling feature extraction block and a feature extraction block. The structure of the feature extraction branch is illustrated in Fig. 3.

Conventional convolutional operations often involve the downsampling of features, leading to information loss. The shallower the layer, the more significant the information loss caused by downsampling. To mitigate the information loss resulting from downsampling, specific convolutional layers within the feature extraction branch are designed to expand the feature dimensions without altering the feature size, achieved by adjusting the convolutional parameters. In the branch, the two convolutional layers following the image input do not perform downsampling, aiming to retain information from shallow features, and the result of the second convolutional layer is exported as the feature for the first scale. The features for the remaining four scales are derived from feature extraction pairs. Moreover, the latter module of the two feature extraction blocks in each pair does not undergo downsampling, effectively delaying the consecutive loss of feature information. The acquisition of scale-specific features through the feature extraction pairs can be represented as follows:

1 $$\begin{aligned} {{F}_{i}}=FE{{B}_{i}}(FEB\_dow{{n}_{i}}({{F}_{i-1}})),i=1,2,3,4, \end{aligned}$$

where $F_i$ represents the *i*th scale feature, $F_0$ is the first scale feature obtained by convolution, $FEB_i$ and $FEB\_down_i$ represent the *i*th feature extraction block and *i*th downsampled feature extraction blocks.

Sobel convolution can introduce rich gradient information into the feature extraction process by calculating the gradient magnitude of the feature, so Sobel convolution is used in the feature extraction block. Sobel convolution can be discretized by the formula as:

2 $$\begin{aligned} \begin{aligned} \text {S(}x,y\text {)=}&\left| {{\Delta }_{x}}f+{{\Delta }_{y}}f \right| \\ =&|(f(x-1,y-1)+2f(x-1,y)+f(x-1, \\&+ y+1))-(f(x+1,y-1)+2f(x+1,y) \\&\times f(x+1,y+1))|+|(f(x-1,y-1)+2f(x,y-1) \\&+f(x+1,y-1))-(f(x-1,y+1) \\&+ 2f(x,y+1)+f(x+1,y+1))|, \\ \end{aligned} \end{aligned}$$

where *S* is the feature after Sobel convolution, *x* and *y* are the coordinates of the pixel on the image respectively, *f* is the feature before Sobel convolution, $\Delta _{x}$ and $\Delta _{y}$ are the Sobel gradient operators in the horizontal and vertical directions respectively.

**Figure 4:**
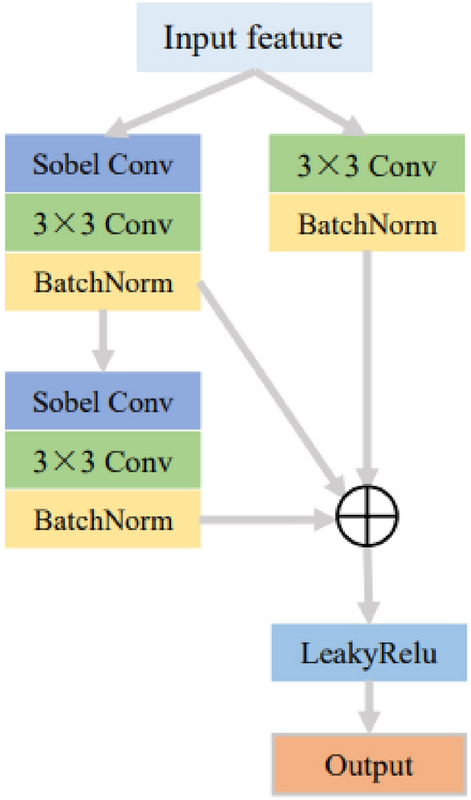
The structure of feature extract block.

The structure of the feature extraction block is depicted in Fig. 4. The features from the input module are divided into two processing branches: the standard convolution and the Sobel convolution. Within the Sobel convolution branch, there are two stages of Sobel convolutions followed by regular convolutions and a skip connection is employed. The inclusion of the skip connection serves the purpose of preventing gradient explosion. An individual feature from the standard convolution processing branch and two features from the Sobel convolution processing branch are combined element-wise. The resultant summation then undergoes processing through the LeakyReLU activation function, yielding the output of this module.

#### Attention-based feature fusion and reconstruction

**Figure 5:**
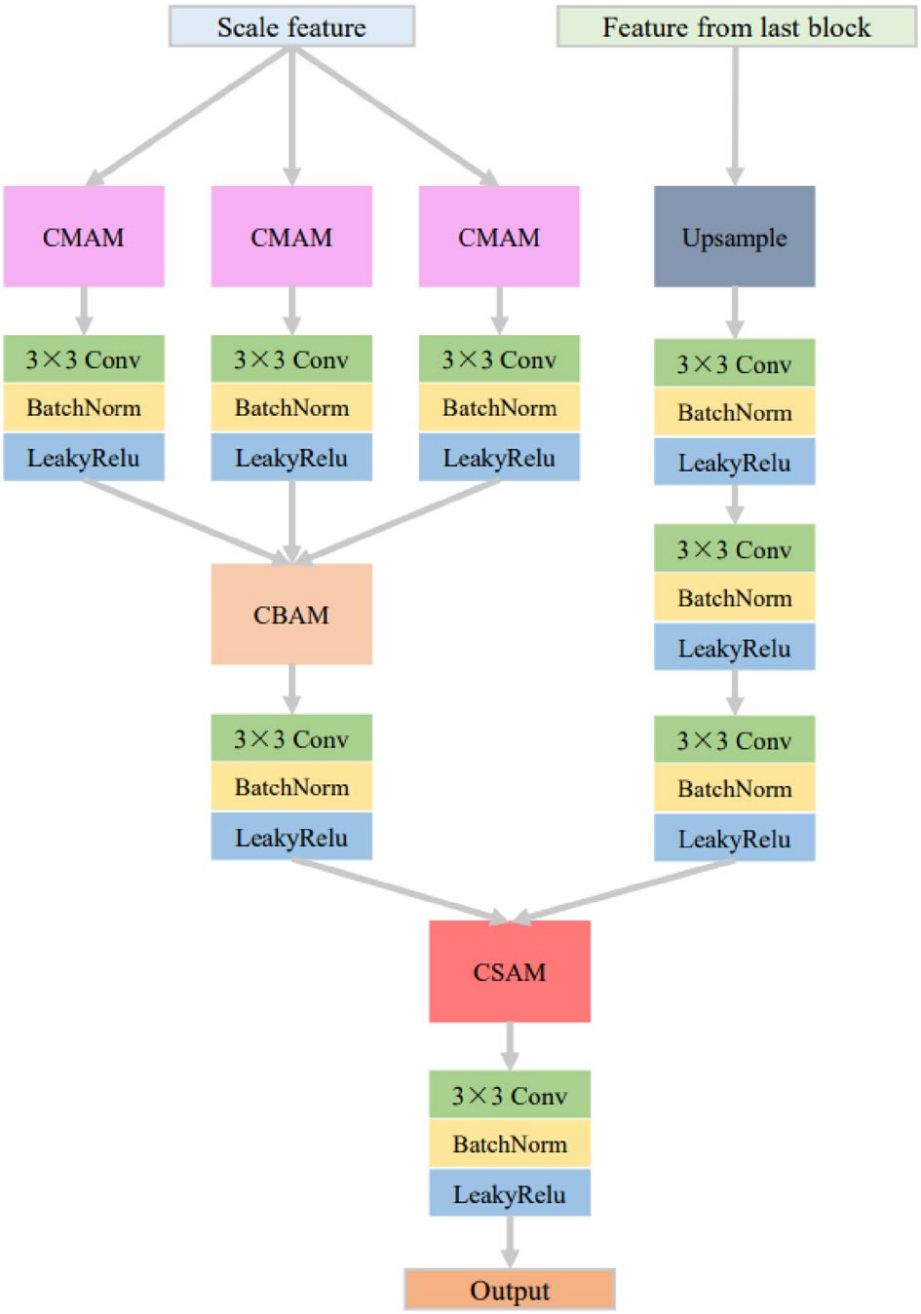
The structure of feature fusion reconstruction module.

The feature fusion and reconstruction network primarily consists of attention-based feature fusion and reconstruction blocks. The structure of the feature fusion and reconstruction block is illustrated in Fig. 5. The module takes as input the image features at the current scale and the features processed by the previous feature fusion and reconstruction block. For the processing of features at the current scale, to fuse the features from two image modalities, the input features are divided into three processing branches, each corresponding to one modality’s branch feature. The image features of the two modalities within each branch are fused using cross-modal attention fusion blocks. Subsequently, each modality branch undergoes a 3 $\times$ 3 convolutional normalization activation process, and the image features between different branches are fused using cross-branch attention fusion blocks. The features from the fused branches are then convolved, normalized, activated, and processed further in the cross-scale fusion reconstruction block. Throughout this process, the convolutional normalization activation between modules aims to introduce mappings and nonlinearity, enhancing feature extraction between modules. Regarding the processing of features at a larger scale, the features are first upsampled to match the size of the features at the current scale. The features are then processed through three consecutive 3 $\times$ 3 convolutions with normalization activations. The gradual reduction of feature dimensions through this step helps mitigate information loss caused by abrupt dimension reduction, as opposed to a direct single-step dimension reduction. The processed features from the larger and current scales are fused in the cross-scale attention fusion module. Similarly, the features from the fused result undergo another round of convolutional normalization activation, yielding the fusion and reconstruction block output. It is important to note that there is no input from features processed at a higher level in the first fusion and reconstruction block. Hence, there is no processing for them or cross-scale feature fusion. The above processing can be represented using equations:

3 $$\begin{aligned} \begin{aligned} {{F}_{so,i}}=&C(CSA{{M}_{i}}(C(CBA{{M}_{i}}(C(CMA{{M}_{i,1}}({{F}_{si,i}})),\\&C(CMA{{M}_{i,2}}({{F}_{si,i}})),C(CMA{{M}_{i,3}}({{F}_{si,i}})))),\\&C(Up({{F}_{so,i-1}})))). \end{aligned} \end{aligned}$$

**Figure 6:**
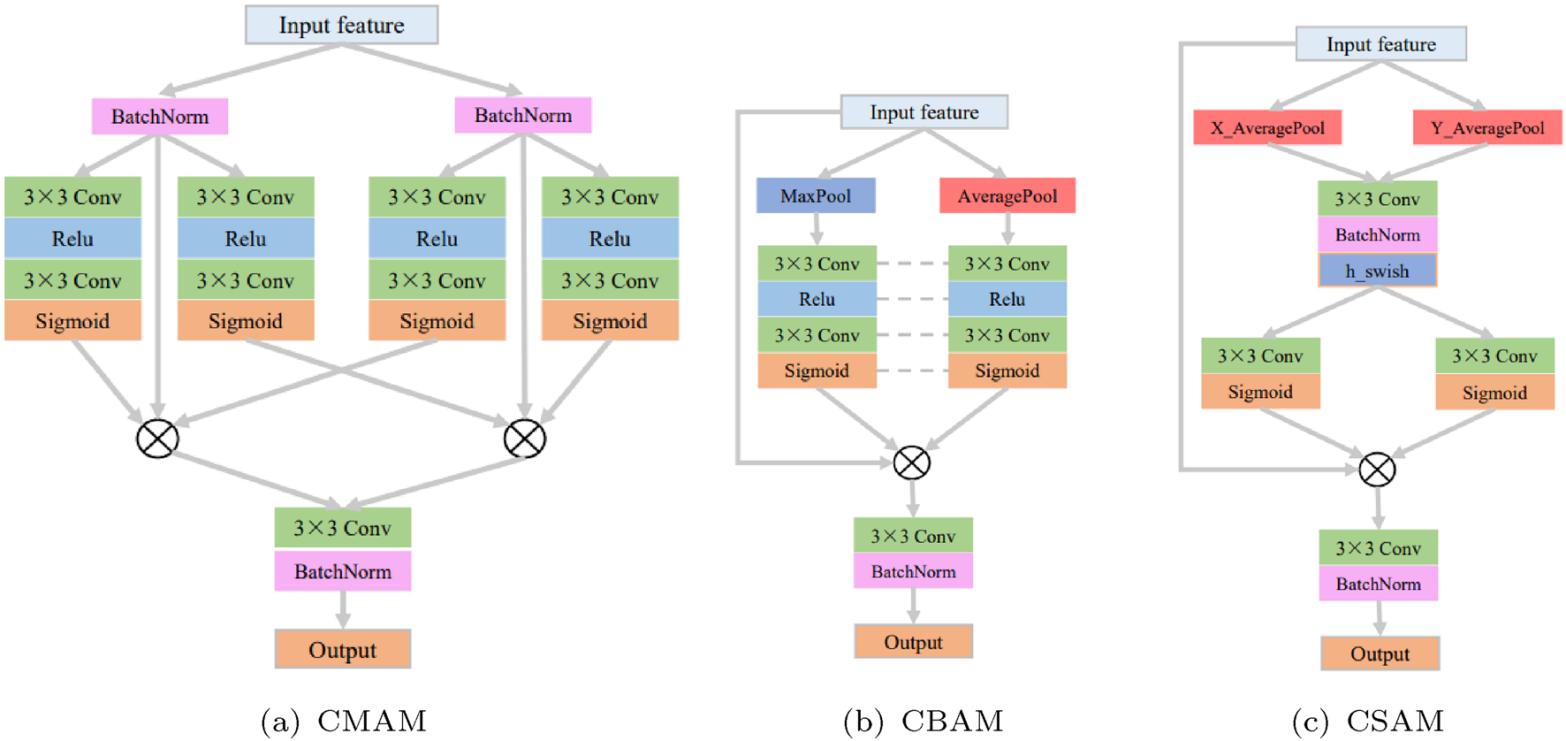
The structure of attention modules.

Figure 6 shows the structure of the three attention fusion modules designed and used in the fusion reconstruction block. From left to right, they are cross-modal attention fusion module, cross-branch attention fusion module and cross-scale attention fusion module.

The purpose of the cross-modal attention fusion block is to facilitate mutual feature interaction between modalities through cross-modal attention, which is why you can observe cross interactions within the structure of the block. The left and right branches in the structure correspond to the processing of features from two modalities. Normalizing input features in the branches helps mitigate the influence of extreme values, thereby enhancing the convergence speed and generalization ability of the network. The construction of attention leverages the sparsity property of the ReLU activation function and the characteristic of the Sigmoid activation function, which maps results between 0 and 1, reflecting the level of attention. The image features from both modalities in the two branches are attended to by attention from their modality as well as the attention from the other modality. The symbol $\otimes$ in the diagram represents attention multiplication. The features modified by attention from both modalities are concatenated and then dimension-reduced by convolutional processing, effectively refining the features and highlighting the attended portions of the features from both modalities. Taking one branch as an example, the application of cross-modal attention can be represented using the following formula:

4 $$\begin{aligned} \begin{aligned}{}&F_{mc,a}=F_{m,a}\cdot (1+M_{m,a}+M_{cm,a}), \\&M_{m,a}=Sigmoid_{a}(Conv_{a}(Relu_{a}(Conv_{a}(F_{m,a})))), \\&M_{cm,a}=Sigmoid_{b}(Conv_{b}(Relu_{b}(Conv_{b}(F_{m,b})))), \end{aligned} \end{aligned}$$

where $F_{m,a}$ represents the input image of the current modality, $F_{mc,a}$ represents the current modality image after applying cross-modality attention, $M_{m,a}$ represents the attention mask for the current modality, $M_{cm,a}$ represents the attention mask for cross-modality, $Conv_{a}$ and $Conv_{b}$ represent convolutional layers in the current modality branch and the other modality branch respectively, $Relu_{a}$ and $Relu_{b}$ represent the ReLU activation function in the current modality branch and the other modality branch respectively, $Sigmoid_{a}$ and $Sigmoid_{b}$ represent the Sigmoid activation function in the current modality branch and the other modality branch respectively.

The cross-branch attention fusion block is based on the convolutional block attention module, with modifications to the utilization of attention and subsequent processing. This fusion module employs both max pooling and average pooling to aggregate feature information across the channels of the branches, utilizing a shared set of attention weights to reduce parameter overhead. After applying attention to the features, a convolutional layer is used to reduce dimensionality, highlighting essential features. The subsequent normalization process serves to stabilize gradients. The application of cross-branch attention can be represented as follows:

5 $$\begin{aligned} \begin{aligned}{}&F_{bc}=BN(Conv(F_{b}\cdot (1+M_{\text{max}}+M_{\text{avg}}))),\\ {}&M_{\text{max}}=Sigmoid(Conv(\text{Re}lu(Conv(Maxpool(F_{b}))))),\\ {}&M_{avg}=Sigmoid(Conv(\text{Re}lu(Conv(Avgpool(F_{b}))))),\end{aligned} \end{aligned}$$

where $F_{b}$ represents the input features, $F_{bc}$ represents the features after cross-branch attention processing, $M_{\text{max}}$ represents the attention mask for max pooling, $M_{\text{avg}}$ represents the attention mask for average pooling, $Maxpool(\cdot )$ represents the max pooling operation, $Avgpool(\cdot )$ represents the average pooling operation, $BN(\cdot )$ represents the batch normalization process.

The cross-branch attention fusion block is built upon the coordinated attention module, with modifications applied to the changes in feature dimensions and post-processing of attention features. Horizontal and vertical average pooling operations are introduced to incorporate spatially contextual information, aiding in the precise localization of position-related changes brought about by scale variations in features from both the current scale and the larger scale. This allows for timely adjustments of relevant attention. The features after attention are similarly dimension-reduced through convolutional operations and normalized. The process of applying cross-scale attention can be represented using the following formula:

6 $$\begin{aligned} \begin{aligned}{}&F_{sc}=BN(Conv(F_{s}\cdot (1+M_{x}+M_{y})))),\\ {}&M_{x}=Sigmoid_{x}(Conv_{x}(hswish(BN(Conv(XAvg(F_{s})))))),\\ {}&M_{y}=Sigmoid_{y}(Conv_{y}(hswish(BN(Conv(YAvg(F_{s})))))),\end{aligned} \end{aligned}$$

where $F_{s}$ represents the input features, $F_{sc}$ represents the features after cross-scale attention processing, $M_{x}$ and $M_{y}$ represent the attention masks for the *x* direction and *y* direction respectively, $XAvg(\cdot )$ and $YAvg(\cdot )$ represent the average pooling for the *x* direction and *y* direction respectively, $hswish(\cdot )$ represents the hsiwsh activation function, $Conv_{x}$ and $Conv_{y}$ represent the independent convolutional layers for the *x* direction branch and *y* direction branch respectively, $Sigmoid_{x}$ and $Sigmoid_{y}$ represent the independent Sigmoid activation functions for the *x* direction branch and *y* direction branch respectively.

### Unsupervised image segmentation networks

The training process of the multimodal medical image fusion network in this approach involves obtaining semantic information through advanced visual tasks to enhance the fusion capability of the network and achieve higher quality fused images. The latter is more suitable for medical imaging scenarios among advanced visual tasks such as image classification, object detection, and image segmentation. Thus, image segmentation is chosen as the source of image semantic information. Most existing image segmentation methods are supervised, imposing high demands on the dataset used for training requiring accurate segmentation labels. Simultaneously, image fusion datasets require images containing two modalities, and these different-modality images need to be registered. Currently, no medical image dataset fulfils image fusion and image segmentation requirements. This issue can be approached by annotating medical image fusion datasets to provide segmentation labels or employing unsupervised image segmentation networks. The former incurs substantial cost, and the resulting methods might lack generality. On the other hand, unsupervised image processing methods are currently a research trend, with some unsupervised approaches capable of achieving results comparable to supervised methods.

In unsupervised image segmentation, we have identified an approach known as “Pixel-wise Feature Clustering Using Invariance and Equivariance”^55^. This unsupervised image segmentation technique employs geometric consistency as an inductive bias to learn the photometric invariance and geometric equivariance of images, facilitating image segmentation without the need for hyperparameter tuning or specific task preprocessing. The method demonstrates robust segmentation results. Within this approach, the alternating use of current feature representations is employed for unsupervised clustering, and the resulting cluster labels are used as pseudo-labels to train feature representations in an iterative manner, ultimately leading to stable outcomes. Moreover, the chosen method for unsupervised image segmentation offers a rational interpretation of its utilization of photometric invariance and geometric equivariance, rooted in sound theory. Photometric invariance entails that pixels in the same position should receive identical labels when there is a minor fluctuation in light intensity of the image, preserving their original division. This concept is manifested in their segmentation method as the feature representations obtained after subjecting each pixel to two distinct photometric transformations should remain consistent. Based on the idea of photometric invariance, clustering pixels transformed under two photometric alterations should ideally be closer to their respective cluster centers and also closer to the cluster centers of the other photometric transformation. Geometric equivariance implies that when an image undergoes geometric transformations like scaling, the resulting cluster segmentation labels should undergo corresponding scaling. The method embodies this principle by applying photometric transformations to both branches and subjecting one branch to a geometric transformation while keeping the other branch invariant, thus creating two distinct geometric forms.

In conclusion, taking into account both the aspects of data considerations and the current landscape of relevant research, the image fusion method proposed in this paper opts for leveraging an unsupervised image fusion network to acquire image semantic information, thereby assisting in the training of the image fusion network. This approach involves utilizing a pixel-wise feature clustering technique incorporating invariance and equivariance principles to segment fused images.

### Design of loss function

The method proposed here defines the loss function of the fusion network from three perspectives, and correspondingly, the final loss function is composed of three components. From the aspect of image content, medical fusion images should strive to incorporate high-intensity information and weak texture details present in the images, such as calcifications or hemorrhagic lesions in brain CT images, along with soft tissue details in MRI images. Regarding image accuracy, the generated fusion image should closely resemble the original two-modal images and not favor one modality over the other. Medical fusion images should encapsulate ample semantic information regarding image semantics, reflecting the fusion network’s understanding of image content. The overall loss function of the fusion network can be represented as follows:

7 $$\begin{aligned} L={{L}_{content}}+{{L}_{similarity}}+{{L}_{semantic}}, \end{aligned}$$

where *L* represents the total loss function, $L\_content$ represents the content loss function, $L\_similarity$ represents the similarity loss function, and $L\_semantic$ represents the semantic loss function.

#### Content loss function

The content loss function measures the content information contained within the fused image. The fusion network, employing the content loss function, iteratively refines the salient features and textural aspects of the fused image, thereby achieving enhancement. This process entails balancing the overall luminosity of the image alongside intricate detail preservation. The content loss function comprises two primary components: intensity loss and texture loss. Its formulation is as follows:

8 $$\begin{aligned} {{L}_{content}}={{L}_{{\text {int}}}}+\alpha {{L}_{texture}}, \end{aligned}$$

where $L\_int$ represents the intensity loss, $L\_texture$ represents the texture loss, and $\alpha$ in the formula is a balance constant with a value of 5.

Intensity loss measures the pixel-by-pixel energy difference between the fused image and the source image and constrains the overall intensity of the fused image. The formula for intensity loss is defined as:

9 $$\begin{aligned} {{L}_{{\text {int}}}}\text {=}\frac{\text {1}}{HW}\left\| {{I}_{f}}-\max ({{I}_{ct}},{{I}_{mri}}) \right\| , \end{aligned}$$

where *H* and *W* are the height and width of the image respectively, $||\cdot ||$ represents the L1 norm calculation, and $max(\cdot )$ represents the element-wise maximum value of the matrix.

Texture loss measures the pixel-by-pixel gradient difference between the fusion image and the source image and reflects the texture difference between the fusion image and the source image. The formula for texture loss is defined as:

10 $$\begin{aligned} {{L}_{texture}}\text {=}\frac{\text {1}}{HW}\left\| \left| \nabla {{I}_{f}} \right| -\max (\left| \nabla {{I}_{ct}} \right| ,\left| \nabla {{I}_{mri}} \right| ) \right\| , \end{aligned}$$

where $\Delta$ is the Sobel gradient operator, which calculates the gradient between pixels, and $|\cdot |$ is the absolute value operation.

The content loss function balances the processing of global features and local details by combining intensity loss and texture loss so that the fused image tends to have rich image content.

#### Similarity loss function

The similarity loss function is grounded in the structural similarity index, where the mean differences in the structural similarity between the fused image and the modalities of the two source images are extracted as the loss. The structural similarity index measures the resemblance between two images by treating them as signals and computing statistical properties such as mean, variance, and covariance of these signals. A comprehensive similarity value is obtained by evaluating the similarity of statistical characteristics between the original and the evaluated image. The structural similarity index effectively characterizes the level of distortion present in an image. The calculation of the structural similarity index is expressed as follows:

11 $$\begin{aligned} \begin{aligned} SSIM(x,y)&=\frac{(2{{\mu }_{x}}{{\mu }_{y}}+{{c}_{1}})(2{{\sigma }_{xy}}+{{c}_{2}})}{(\mu _{x}^{2}+\mu _{y}^{2}+{{c}_{1}})(\sigma _{x}^{2}+\sigma _{y}^{2}+{{c}_{2}})}, \\ {{c}_{1}}&={{(0.01L)}^{2}}, \\ {{c}_{2}}&={{(0.03L)}^{2}}, \\ \end{aligned} \end{aligned}$$

where ${{\mu }_{x}}$ and ${{\mu }_{y}}$ represent the average value of image *x* and image *y* respectively, $\sigma _{x}^ {2}$ and $\sigma _{y}^{2}$ represent the variance of image *x* and image *y* respectively, and ${{\sigma }_{xy}}$ represent the covariance of image and image, ${{c}_{1}}$ and ${{c}_{2}}$ are stability constants, and *L* is the dynamic range of pixel values.

The value range of structural similarity is $-1$ to 1, and when the two images are completely consistent, the value is 1. The similarity loss is expressed as:

12 $$\begin{aligned} {{L}_{similarity}}=\text {1}-\frac{SSIM({{I}_{f}},{{I}_{ct}})+SSIM({{I}_{f}},{{I}_{mri}})}{2}, \end{aligned}$$

where ${{I}_{f}}$ represents the fused image, ${{I}_{ct}}$ represents the original CT image, and ${{I}_{mri}}$ represents the original MRI image.

#### Semantic loss function

The semantic loss function of the fused texture is also the segmentation loss of the image segmentation network, which reflects the loss of image semantic information in the image fusion network and the loss of segmentation difference in the unsupervised image segmentation network. The definition of this loss function is based on the photometric invariance and geometric invariance of the unsupervised image segmentation network used, consisting of an intra-view loss and an inter-view loss for unsupervised clustering. In the segmentation network, each fused image ${{x}_{i}}$ is applied with two random image transformations $P_{i}^{(1)}$ and $P_{i}^{( 2)}$, generate two feature vectors $z_{ip}^{(1)}$ and $z_{ip}^{(2)}$ for each pixel *p* in the image ${{x}_{i}}$ by segmenting the feature extraction network $\theta$:

13 $$\begin{aligned} \begin{aligned} z_{ip}^{(1)}&={{f}_{\theta }}(P_{i}^{(1)}({{x}_{i}}))[p], \\ z_{ip}^{(2)}&={{f}_{\theta }}(P_{i}^{(2)}({{x}_{i}}))[p]. \\ \end{aligned} \end{aligned}$$

Subsequently, two sets of pseudo-labels and cluster centers are obtained by performing independent clustering on the two feature views:

14 $$\begin{aligned} \begin{aligned} {{y}^{(1)}},{{\mu }^{(1)}}&=\arg \underset{y,\mu }{\mathop {\min }}\,{{\sum \limits _{i,p}{\left\| z_{ip}^{(1)}-{{\mu }_{{{y}_{ip}}}} \right\| }}^{2}}, \\ {{y}^{(2)}},{{\mu }^{(2)}}&=\arg \underset{y,\mu }{\mathop {\min }}\,{{\sum \limits _{i,p}{\left\| z_{ip}^{(2)}-{{\mu }_{{{y}_{ip}}}} \right\| }}^{2}}, \\ \end{aligned} \end{aligned}$$

where *y* is the corresponding pseudo-label, and $\mu$ represents the corresponding cluster center.

15 $$\begin{aligned} {{L}_{clust}}({{f}_{\theta }}({{x}_{i}})[p],{{y}_{ip}},\mu )=-\log \frac{{{e}^{-d({{f}_{\theta }}({{x}_{i}})[p],{{\mu }_{{{y}_{ip}}}})}}}{\sum \nolimits _{l}{{{e}^{-d({{f}_{\theta }}({{x}_{i}})[p],{{\mu }_{l}})}}}}, \end{aligned}$$

where $d(\cdot ,\cdot )$ represents the cosine distance.

The feature vectors from both views need to correspond to the corresponding cluster labels, thus defining the in-view loss:

16 $$\begin{aligned} \begin{aligned} {{L}_{within}}=&\sum \limits _{i,p}{{{L}_{clust}}(z_{ip}^{(1)},y_{ip}^{(1)},{{\mu }^{(1)}})} \\&+\sum \limits _{i,p}{{{L}_{clust}}(z_{ip}^{(2)},y_{ip}^{(2)},{{\mu }^{(2)}})}. \end{aligned} \end{aligned}$$

Corresponding to different image photometric transformations, the eigenvectors between two views need to be consistent with another cluster label, thus defining the semantic loss between views:

17 $$\begin{aligned} \begin{aligned} {{L}_{cross}}=&\sum \limits _{i,p}{{{L}_{clust}}(z_{ip}^{(1)},y_{ip}^{(2)},{{\mu }^{(2)}})} \\&+\sum \limits _{i,p}{{{L}_{clust}}(z_{ip}^{(2)},y_{ip}^{(1)},{{\mu }^{(1)}})}. \end{aligned} \end{aligned}$$

Finally, the semantic loss function is the sum of the intra-view loss and the inter-view loss, expressed as:

18 $$\begin{aligned} {{L}_{semantic}}={{L}_{within}}+{{L}_{cross}}. \end{aligned}$$

## Experiments

In this section, we provide an elucidation of the experimental setup and implementation particulars. Subsequently, we showcase the superiority of the proposed methodology DUSMIF through a comparative analysis of the experimental outcomes. Finally, we substantiate the effectiveness of the devised approach through ablation experiments, confirming the integration of semantic information, the utility of multi-branch and multi-scale feature extraction, and the efficacy of attention mechanisms.

### Settings

To comprehensively assess the proposed approach and validate its efficacy and reliability, this study conducted extensive quantitative and qualitative experiments on the Harvard Whole Brain Dataset. The proposed method was rigorously compared against nine state-of-the-art methodologies. For each of these methodologies, the implementation utilized was the publicly available code provided by the respective authors.

1. Dual-Discriminator Conditional Generative Adversarial Network (DDcGAN)^21^.
2. Multiscale Residual Pyramid Attention Network (MSRPAN)^27^.
3. General Unsupervised Image Fusion Network based on Memory Unit (MUFusion)^18^.
4. Coupled GAN with Relativistic Discriminators (RCGAN)^22^.
5. Unified Unsupervised Image Fusion Network (U2Fusion)^19^.
6. Enhanced Medical Image Fusion Network (EMFusion)^20^.
7. Multimodal Medical Image Fusion via Multiscale Adaptive Transformer (MATR)^28^.
8. Multi-Scale DenseNet for Medical Image Fusion (MSDNet)^56^.
9. Image Fusion Network based on Proportional Maintenance of Gradient and Intensity (PMGI)^57^.
10. A functional–anatomical transformer for medical image fusion (FATFusion)^58^.
11. A real-time medical image fusion method guided by detail information (LRFNet)^59^.
12. Joint Residual Swin Transformer and Multiscale CNN for Unsupervised Multimodal Medical Image Fusion (MRSCFusion)^60^.

Eight statistical evaluation metrics were employed to quantitatively assess the proposed method and the fusion results of the abovementioned techniques. These metrics include Peak Signal-to-Noise Ratio (PSNR), Average Gradient (AG), Spatial Frequency (SF), Visual Information Fidelity (VIF), Noise Assessment-Based Fusion (Nabf), Structural Similarity Index (SSIM), Multi-Scale Structural Similarity (MSSSIM), and Gradient-Based Fusion Performance (Qabf). PSNR quantifies the peak signal-to-noise ratio between the original and fused images, indicating the ratio of information to noise. SF indicates pixel variation rate within an image, where higher spatial frequencies correspond to more explicit images. AG describes the sharpness of the image based on gradient information, with larger values denoting a better representation of details. VIF is an objective quality assessment metric used to evaluate the impact of image distortion on visual perception. SSIM measures the degree of similarity between two images, assessing structural, contrast, and luminance factors rather than traditional pixel differences. MSSSIM extends this by considering multiple levels of structural similarity, aligning more closely with the perception of the human visual system. Qabf evaluates the presentation of salient information in the fusion image through local metrics. Nabf represents the ratio of artificially introduced noise during the fusion process. These metrics collectively offer a comprehensive evaluation of the proposed method and its fusion outcomes, ensuring a robust assessment of effectiveness and quality.

### Implementation details

The experiment utilized data from the publicly available Harvard Whole Brain Medical Dataset (AANLIBb), which offers co-registered multi-modal brain medical images spanning various pathological conditions. The input images to the proposed fusion algorithm were sized at 256 $\times$ 256, with the output images at 256 $\times$ 256 dimensions. The fusion network underwent a training epoch of 20, while the segmentation network underwent a single training epoch of 10. The fusion and segmentation networks were alternately trained for five cycles. The nine methods used for comparison were also trained on the same dataset split. The parameters used in these methods were sourced from publicly available of the original authors code. All experiments were conducted on a PowerEdge-R750xa server with an Intel Xeon Gold 6326 CPU, 512 GB of RAM, and an NVIDIA A100-PCIe 40 GB GPU. This standardized experimental setup ensures consistency and fairness in evaluating the proposed method against the state-of-the-art alternatives on the Harvard Whole Brain Dataset.

### CT-MRI fusion comparison experiment

In order to fully evaluate the fusion performance, the proposed method is used to fuse CT and MRI images in the AANLIB dataset and compared with the other algorithms.

#### Qualitative analysis

**Figure 7:**
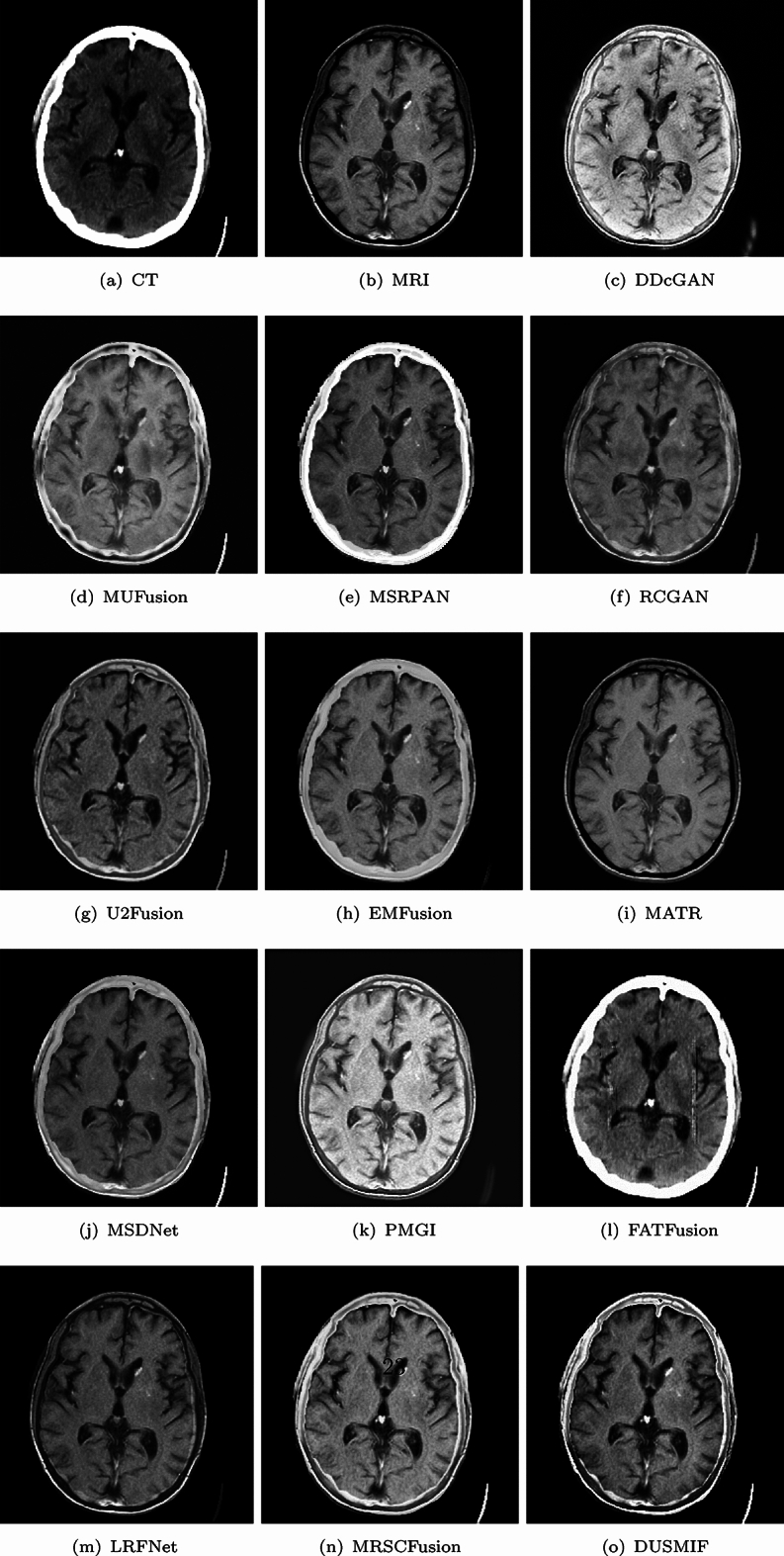
The first comparative display of CT-MRI medical image fusion.

In Fig. 7, a brain image of a patient with cerebral toxoplasmosis and encephalitis is presented. The CT image reveals calcifications in the dura mater and frontal lobe, while the MRI image displays multiple localized lesions in the basal ganglia. Among all the compared methods, the fused brain region in the DDcGAN-generated fusion image appears excessively bright and exhibits slight artifacts, making it difficult to discern the lesions. The fusion image generated by FATFusion is predominantly influenced by the CT images, and it does not show significant content related to the MRI images. It can be seen that the method is not adapted for the fusion of CT-MRI images. The fused image from PMGI has a grayish background with overly bright brain structures, which also hampers the preservation of image details. In the fusion images generated by MSRPAN, EMFusion and MSDNet, the regions between the skull and frontal lobe are dominated by the CT image, leading to inadequate visualization of the calcifications in the frontal lobe. In contrast, in the MATR and LRFNet generated fusion image, the areas between the skull and frontal lobe are predominantly influenced by the MRI image, obscuring the visual representation of calcified deposits.

**Figure 8:**
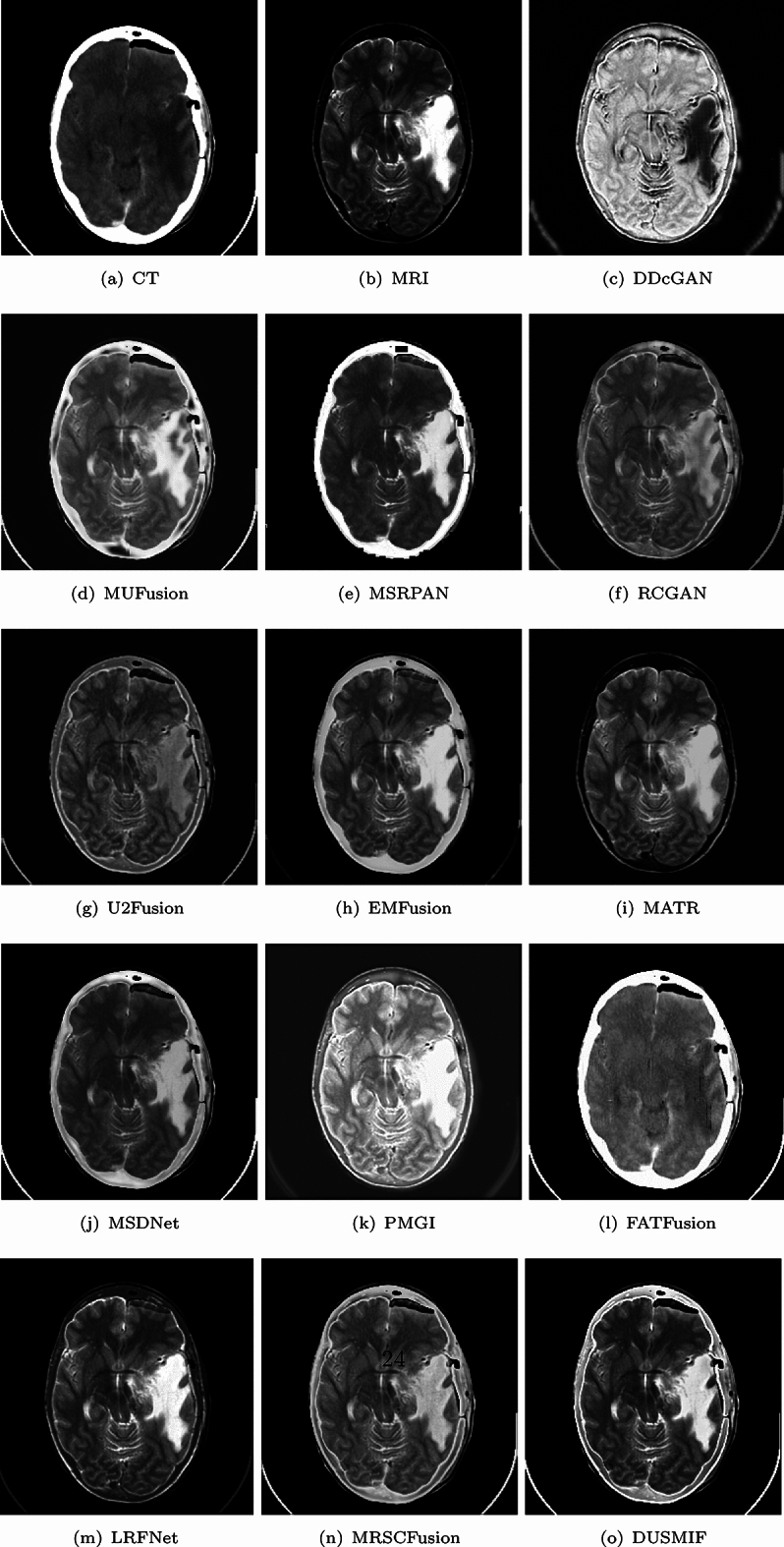
The second comparative display of CT-MRI medical image fusion.

Among the fusion images produced by MUFusion, RCGAN, U2Fusion and DUSMIF, the calcified deposits near the skull are visible, with DUSMIF exhibiting the most precise representation. Additionally, the high-signal basal ganglia lesion in the MRI image is not pronounced in DDcGAN and PMGI due to increased baseline brightness. While it is discernible in methods like MUFusion, U2Fusion and MSDNet, the contrast is lower. In contrast, the outcome of the proposed method displays a higher contrast between the localized lesions and surrounding tissues, enabling accurate observation. In this case, the fused image generated by the proposed method possesses appropriate brightness, strong contrast, and apparent visual effects, making it the most suitable among the compared methods for accurate observation.

In Fig. 8, a brain image of a patient with metastatic bronchogenic carcinoma after decompressive surgery is presented. The CT image displays the cranial opening point, while the MRI image depicts the tumor as a high-signal mass in the temporal area, presenting as a large tumor with surrounding edema and cystic components. Among all the fusion images of the compared methods, the DDcGAN-generated fusion image excessively brightens the tissue areas and darkens the pathological regions, resulting in a significant loss of detail and considerable artifacts. The fused images of FATFusion are also primarily presented in the style of CT images, confirming the previous observation. The fusion images generated by LRFNet do not preserve the cranial bones well from the CT images. The fused image from PMGI also suffers from overly bright brain regions, though slightly better than DDcGAN. The fusion images of RCGAN, U2Fusion and MSRCFusion exhibit an overall darkened appearance, leading to low image contrast. Similarly, the fusion images from EMFusion and MSDNet suffer from a slightly darkened appearance and lower contrast, but they fare better than the previous three methods. Moreover, EMFusion preserves more image details than MSDNet. The fusion image generated by MATR faces contrast reduction issues, and the skull region is not depicted, hindering observation of the cranial opening. The fusion image from MSRPAN retains fewer details in brain tissue. MUFusion and the proposed method demonstrate better handling of image contrast and detail preservation. The proposed method exhibits superior contrast compared to MUFusion. When comparing the fusion results of the proposed method with other methods on this example image, the proposed method stands out for its comprehensive detail preservation and high image contrast.

#### Quantitative analysis

The numerical results of various evaluation metrics are presented in Table 1. Red indicates the best result for each metric, blue indicates the second best, and green indicates the third best. It can be observed that the proposed method achieves the best results in terms of AG, Qabf, SF, SSIM, and MSSSIM metrics. This suggests that the fused images generated by the proposed method contain more image details and transfer the most prominent local information from the source images to the fused image. Additionally, the fused images generated by the proposed method are the clearest among all the compared methods. Furthermore, the proposed method achieves the best performance in terms of SSIM and MSSSIM metrics, indicating that the fused images maintain the most similarity in image structure and multi-level structures with the two source images. While the proposed medical image fusion method does not achieve a top-three position in PSNR, VIF and Nabf metrics, its results closely follow those in the top three positions, with only a minor gap. This indicates that the proposed method does not exhibit significant shortcomings and remains competitive regarding these metrics.

**Table 1:**
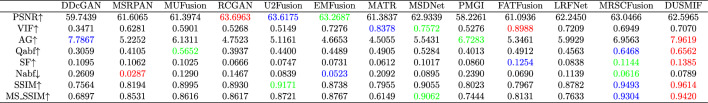
Metrics results of CT-MRI medical images fusion.

**Figure 9:**
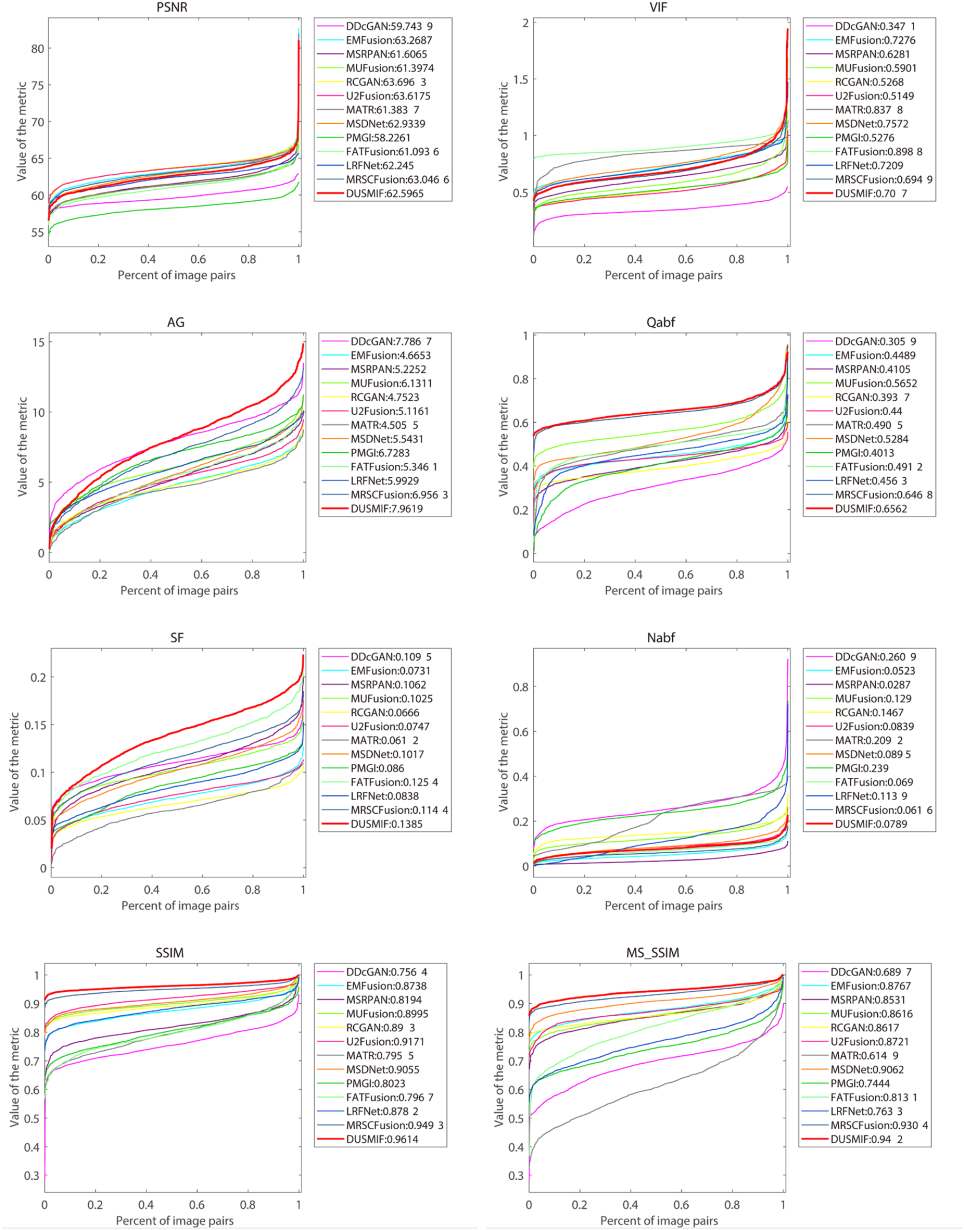
Quantitative comparison of proposed method with state-of-the-art methods in CT-MRI modality.

The quantified curve results for the eight evaluation metrics on the test images of the dataset are presented in Fig. 9. The image effectively demonstrates an overview of the performance of the fusion algorithm across all test images. In the metrics where the proposed method achieves the best results (Qabf, SF, SSIM, MSSSIM), the curves representing these metrics consistently lie above the curves representing other methods. This indicates that the proposed method outperforms other methods across all test images for these metrics. In the AG curve, the curve representing the proposed method is slightly below the curve for DDcGAN around the position of approximately 0.4. However, beyond this point, the curve of the proposed method surpasses that of DDcGAN. This suggests that the results of the proposed method are inferior to DDcGAN in this metric for a small subset of images but superior for the majority of images. Regarding the PSNR and VIF curves, the curves of the proposed method lie in a moderately better position, aligning with the conclusions drawn from the numerical results.

### PET-MRI fusion comparison experiment

In order to fully evaluate the fusion performance, the proposed method also is used to fuse PET-MRI images in the AANLIB dataset and compared with the other nine algorithms.

#### Qualitative analysis

**Figure 10:**
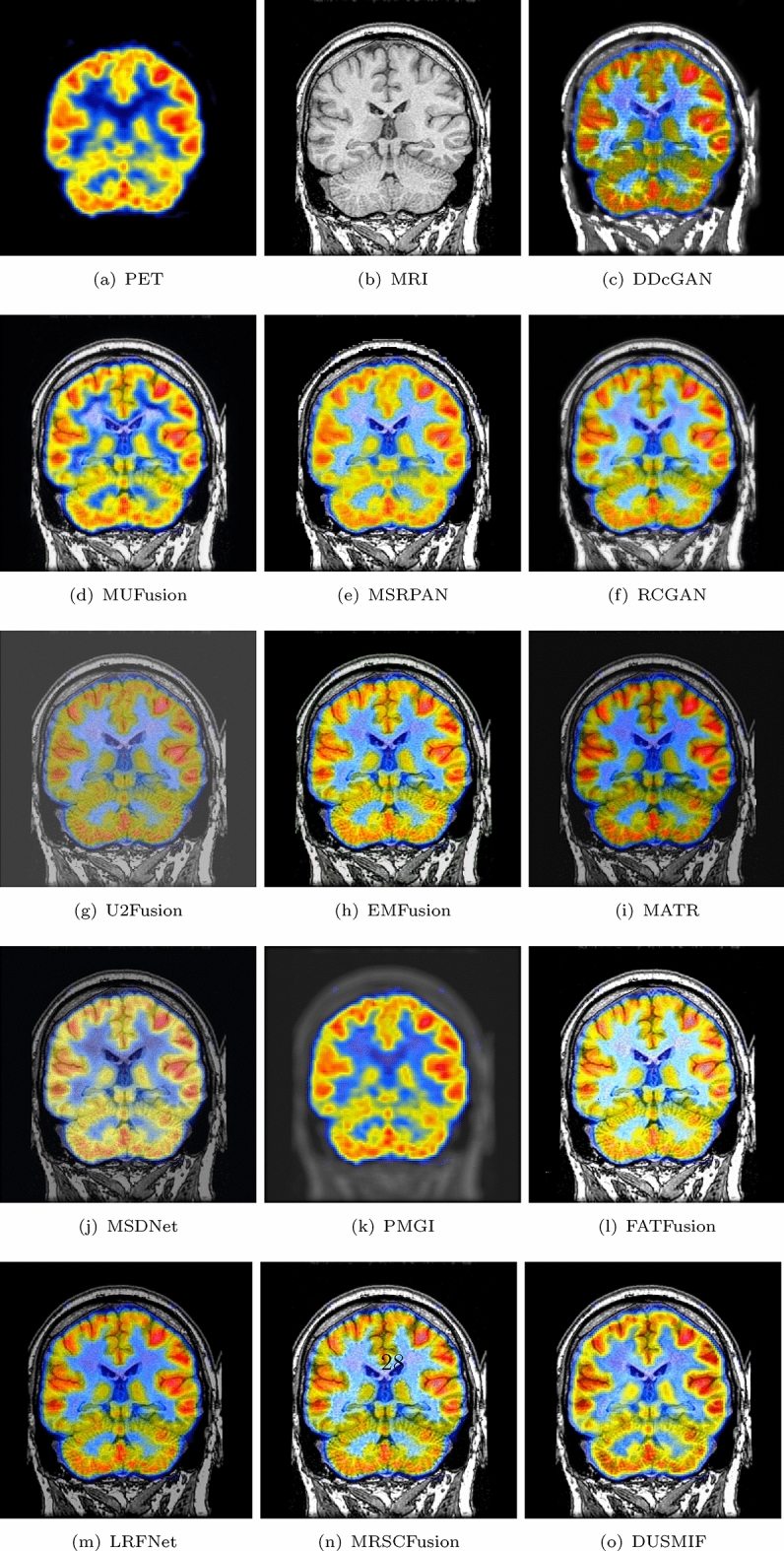
Comparative display of PET-MRI medical image fusion.

In Fig. 10, a brain image of a patient with herpes encephalitis is presented. PET images employ positron-emitting radiotracers to reflect changes in metabolism by measuring the tracer’s uptake in lesions, providing biological metabolic information for clinical purposes. MRI images utilize nuclear magnetic resonance principles to produce images with excellent soft tissue contrast. Among all the fusion images of the compared methods, the U2Fusion-generated fusion image appears desaturated, which hinders image interpretation. In the fusion images generated by FATFusion, there are noticeable changes in the color intensity, which do not accurately reflect the information from the original PET images. On the other hand, the images generated by LRFNet are generally darker, which may affect the observation process. The fused image from PMGI exhibits unclear content from the MRI image, making it challenging to observe brain tissue effectively. The fused image generated by MSDNet suffers from low saturation and both the DDcGAN and MATR fusion images display darkened regions where metabolic activity is expected. Additionally, the fusion image generated by MSRPAN lacks comprehensive preservation of brain tissue details. The remaining methods, MUFusion, RCGAN, EMFusion and DUSMIF, produce similar fusion image results, performing well in contrast, brightness and texture detail preservation.

#### Quantitative analysis

The numerical results of various evaluation metrics are presented in Table 2. Red indicates the best result for each metric, blue indicates the second best, and green indicates the third best. Notably, the proposed method achieves the best results in SF and MSSSIM metrics and the second-best results in SSIM metric. This implies that the fused images generated by the proposed method contain rich image details, transferring the most detail and texture from the source images to the fused image compared to other methods. The generated fused images also exhibit excellent visual effects compared to the other methods. Furthermore, the proposed method achieves the best performance in terms of the SSIM metric and the second-best performance in the MSSSIM metric. This highlights that the fused images maintain the highest structural similarity at both image and multi-level structures with the two source images. While the proposed medical image fusion method does not secure a top-three position in PSNR, VIF, Qabf and Nabf metrics, its results closely follow those in the top three positions with only a minor gap. This indicates that the proposed method remains competitive and does not exhibit significant shortcomings in these metrics.

**Table 2:**
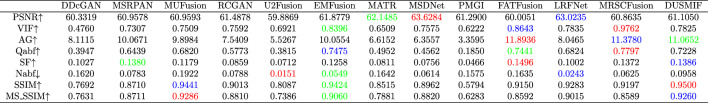
Metrics results of PET-MRI medical image fusion.

**Figure 11:**
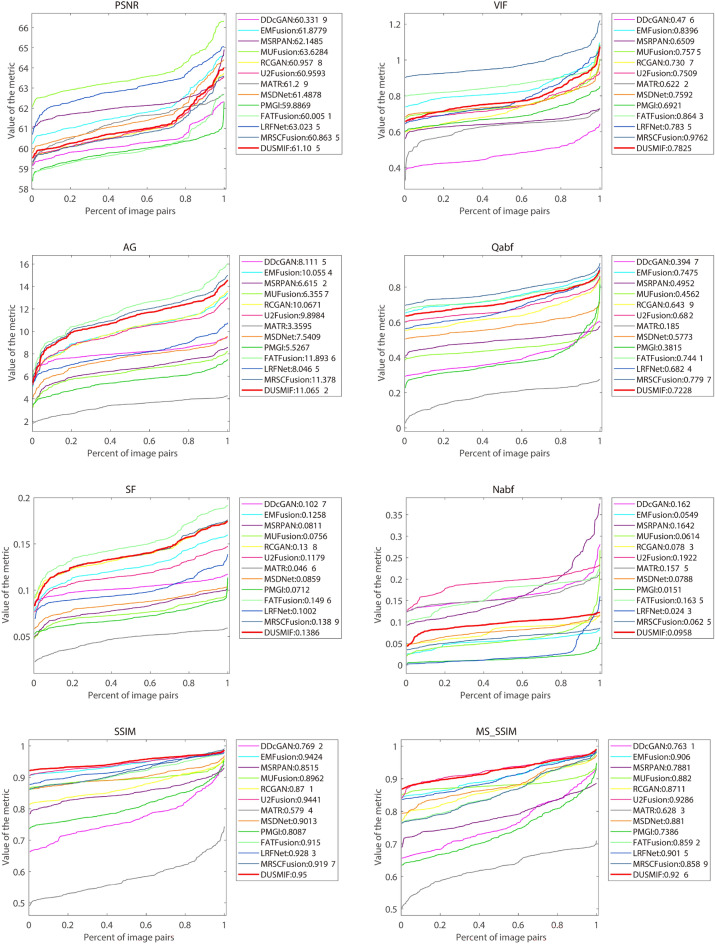
Quantitative comparison of proposed method with state-of-the-art methods in PET-MRI modality.

The quantified curve results of the eight evaluation metrics on testing images are depicted in Fig. 11, effectively illustrating the overall performance of the fusion algorithm across all test images. In the three metrics, AG, SF and SSIM, where the proposed method achieves the optimal results, its curve consistently surpasses the curves representing other methods throughout most intervals. This observation underscores the superior performance of the proposed method over others in the majority of the test images. Regarding the quantified curves for the VIF, Qabf and MSSSIM metrics, the curves of the proposed method are inferior to those that excel in each respective metric. Particularly in the case of the MSSSIM metric, the proposed method’s curve closely aligns with that of the optimal method, underscoring its commendable performance in these metrics and validating the conclusions drawn from the numerical results. In contrast, the quantified curves for the PSNR and Nabf metrics position the performance of the proposed method in a moderate range, in line with the numerical results.

### Ablation study

#### Semantic loss analysis

The proposed approach aims to enhance the quality of fused image results by incorporating a semantic loss augmented fusion network. In order to validate the effectiveness of the semantic loss, an ablation experiment involving semantic loss was devised. In this experiment, both the segmentation network and the segmentation auxiliary loss were removed from the proposed method, and the modified network was trained using identical parameters and datasets. The trained model was then employed to fuse images, and the resulting metrics were computed and compared with the original method.

**Table 3:** Metric results on semantic ablation study.

	Without semantic	With semantic
PSNR$\uparrow$	62.4244	62.5965
VIF$\uparrow$	0.6950	0.7070
AG$\uparrow$	7.9239	7.9619
Qabf$\uparrow$	0.6457	0.6562
SF$\uparrow$	0.1466	0.1385
Nabf$\downarrow$	0.0906	0.0789
SSIM$\uparrow$	0.9552	0.9614
MSSSIM$\uparrow$	0.9440	0.9420

The semantic loss ablation experiment results are presented in Table 3. The method incorporating semantic loss demonstrates superior performance in six evaluation metrics, including PSNR, VIF and AG, compared to the method without semantic loss. The experimental outcomes underscore the effectiveness of introducing semantic loss in enhancing the performance of the image fusion model.

#### Attention mechanism analysis

The proposed approach integrates a multi-attention mechanism into the feature fusion reconstruction module. Cross-modal attention is employed for fusing image features across different modalities, cross-branch attention is utilized for fusing image features across different branches, and cross-scale attention is employed for fusing features across different scales. An ablation experiment involving attention mechanisms was conducted to verify the effectiveness of multi-attention mechanism. In this experiment, the attention mechanisms within the feature fusion reconstruction block were removed, and the modified network was trained using identical parameters and datasets. The trained model was then employed to fuse images, and the resulting metrics were computed and compared with the original method.

**Table 4:** Metric results on attention ablation study.

	Without attention	With attention
PSNR$\uparrow$	62.3452	62.5965
VIF$\uparrow$	0.7202	0.7070
AG$\uparrow$	7.7973	7.9619
Qabf$\uparrow$	0.6453	0.6562
SF$\uparrow$	0.1340	0.1385
Nabf$\downarrow$	0.0776	0.0789
SSIM$\uparrow$	0.9581	0.9614
MSSSIM$\uparrow$	0.9418	0.9420

The attention mechanism ablation experiment results are presented in Table 4. The method employing attention mechanisms exhibits superior performance in six evaluation metrics, including PSNR, AG and Qabf, compared to the method without attention mechanisms. The experimental outcomes highlight the role of attention mechanisms in the fusion network, acting as effective filters for selecting meaningful features and thereby enhancing the capabilities of the image fusion model.

#### Multi-scale and multi-branch feature extraction analysis

The proposed approach employs a multi-scale multi-branch feature extraction network to extract features from source images, providing abundant image features for subsequent feature fusion and reconstruction. An ablation experiment targeting this aspect was designed to validate the effectiveness of the multi-branch and multi-scale feature extraction network. In this experiment, four conditions were tested, namely: without multiscale or multibranch, with multiscale but without multibranch, without multiscale but with multibranch, and with both multiscale and multibranch. Adjustments were made to the remaining parts of the network in each condition to match the feature extraction network accordingly. The modified networks were trained using the same training parameters and dataset, and the trained models were then used for image fusion. The resulting metrics were computed and compared with the original method.

**Table 5:** Metric results on scale and branch ablation study.

Multi-scale	Multi-branch	PSNR$\uparrow$	VIF$\uparrow$	AG$\uparrow$	Qabf$\uparrow$	SF$\uparrow$	Nabf$\downarrow$	SSIM$\uparrow$	MSSSIM$\uparrow$
		61.0996	0.3412	4.8711	0.2168	0.0606	0.1165	0.6392	0.6167
$\checkmark$		62.5107	0.6856	7.7747	0.6305	0.1355	0.0746	0.9566	0.9418
	$\checkmark$	61.2406	0.3609	5.2542	0.2615	0.0740	0.1122	0.6497	0.6342
$\checkmark$	$\checkmark$	62.5965	0.7070	7.9619	0.6562	0.1385	0.0789	0.9614	0.9420

The multi-scale multi-branch feature extraction ablation experiment results are presented in Table 5. The single-scale feature extraction network exhibited poor results. The multi-scale multi-branch feature extraction method demonstrates superior performance in seven evaluation metrics, including PSNR, VIF and AG, compared to single-branch feature extraction. The experimental outcomes underscore the effectiveness of multi-scale multi-branch feature extraction in enhancing the performance of the image fusion model.

## Conclusion

This paper addresses the shortcomings in existing deep neural network-based medical image fusion techniques, particularly the inadequate utilization of semantic information and insufficient feature extraction. To overcome these issues, a novel approach is proposed. Firstly, semantic information is acquired through an image segmentation task and introduced into the medical image fusion network via a segmentation loss function. This incorporation enriches the fusion process with meaningful context. Simultaneously, a multi-branch and multi-scale parallel feature extraction network is devised. This network complements and refines features through different branches, enhancing their quality. Furthermore, a feature fusion and reconstruction network is developed, leveraging multiple attention mechanisms. These attention mechanisms correspond to distinct fusion processes, allowing for diverse and context-aware fusion. To optimize the fusion outcome, a comprehensive fusion loss function is formulated. This loss function comprises luminance loss, texture loss, similarity loss, and semantic loss, collectively enhancing the capability of the network to fuse intricate image details. Experimental results demonstrate that, in comparison to existing medical image fusion methods, our proposed approach exhibits superior performance across objective metrics and subjective evaluations. Ablation experiments further validate the effectiveness of each component within the proposed method.

## Data Availability

The datasets used and/or analysed during the current study available from the corresponding author on reasonable request.

## References

[1] Li, Y. *et al.* A dual attention-guided 3d convolution network for automatic segmentation of prostate and tumor. *Biomed. Signal Process. Control* **85**(104755), 104755. 10.1016/j.bspc.2023.104755 (2023). 10.1016/j.bspc.2023.104755

[2] Cui, Z., Zhang, G. & Wu, J. Medical image fusion based on wavelet transform and independent component analysis. In *2009 International Joint Conference on Artificial Intelligence* (IEEE, 2009).

[3] Wang, H.-Q. & Xing, H. Multi-mode medical image fusion algorithm based on principal component analysis. In *2009 International Symposium on Computer Network and Multimedia Technology* (IEEE, 2009).

[4] Ramya, H. R. & Sujatha, B. K. Fine grained medical image fusion using type-2 fuzzy logic. *Indones. J. Electric. Eng. Comput. Sci.* **14**(2), 999. 10.11591/ijeecs.v14.i2.pp999-1011 (2019). 10.11591/ijeecs.v14.i2.pp999-1011

[5] Zhao, F., Xu, G. & Zhao, W. CT and MR image fusion based on adaptive structure decomposition. *IEEE Access Pract. Innov. Open Solut.* **7**, 44002–44009. 10.1109/access.2019.2908378 (2019). 10.1109/access.2019.2908378

[6] Ch, M. M. I., Ghafoor, A., Bakhshi, A. D. & Saghir, N. J. Medical image fusion using non subsampled contourlet transform and iterative joint filter. *Multimedia Tools Appl.* **81**(3), 4495–4509. 10.1007/s11042-021-11753-8 (2022). 10.1007/s11042-021-11753-8

[7] Li, B. *et al.* Medical image fusion method based on coupled neural p systems in nonsubsampled shearlet transform domain. *Int. J. Neural Syst.* **31**(1), 2050050. 10.1142/S0129065720500501 (2021). 32808852 10.1142/S0129065720500501

[8] Shabanzade, F. & Ghassemian, H. Combination of wavelet and contourlet transforms for pet and MRI image fusion. In *2017 Artificial Intelligence and Signal Processing Conference (AISP)* (IEEE, 2017)

[9] Prakash, O., Park, C. M., Khare, A., Jeon, M. & Gwak, J. Multiscale fusion of multimodal medical images using lifting scheme based biorthogonal wavelet transform. *Optik* **182**, 995–1014. 10.1016/j.ijleo.2018.12.028 (2019). 10.1016/j.ijleo.2018.12.028

[10] Bhateja, V., Krishn, A., Patel, H. & Sahu, A. Medical image fusion in wavelet and ridgelet domains: A comparative evaluation. *Int. J. Rough Sets Data Anal.* **2**(2), 78–91. 10.4018/ijrsda.2015070105 (2015). 10.4018/ijrsda.2015070105

[11] Mathiyalagan, P.: Multi-modal medical image fusion using curvelet algorithm. In *2018 International Conference on Advances in Computing, Communications and Informatics (ICACCI)* (IEEE, 2018)

[12] Kaur, G., Singh, S. & Vig, R. Medical fusion framework using discrete fractional wavelets and non-subsampled directional filter banks. *IET Image Proc.* **14**(4), 658–667. 10.1049/iet-ipr.2019.0948 (2020). 10.1049/iet-ipr.2019.0948

[13] Ramakrishnan, V. & Pete, D. J. Non subsampled shearlet transform based fusion of multiple exposure images. *SN Comput. Sci.* **1**(6), 4. 10.1007/s42979-020-00343-4 (2020). 10.1007/s42979-020-00343-4

[14] Shilpa, S., Ragesh Rajan, M., Asha, C. S. & Shyam, L. Enhanced jaya optimization based medical image fusion in adaptive non subsampled shearlet transform domain. *Eng. Sci. Technol. Int. J.* **35**(101245), 101245. 10.1016/j.jestch.2022.101245 (2022). 10.1016/j.jestch.2022.101245

[15] Gai, D., Shen, X., Chen, H., Xie, Z. & Su, P. Medical image fusion using the pcnn based on iqpso in nsst domain. *IET Image Proc.* **14**(9), 1870–1880. 10.1049/iet-ipr.2020.0040 (2020). 10.1049/iet-ipr.2020.0040

[16] Vanitha, K., Satyanarayana, D. & Prasad, M. N. G. Multi-modal medical image fusion algorithm based on spatial frequency motivated pa-pcnn in the nsst domain. *Curr. Med. Imaging Rev.* **17**(5), 634–643. 10.2174/1573405616666201118123220 (2021). 10.2174/1573405616666201118123220 33213329

[17] Koteswara Rao, K. & Veera Swamy, K. Multimodal medical image fusion using residual network 50 in non subsampled contourlet transform. *Imaging Sci. J.* **718**, 1–14. 10.1080/13682199.2023.2175426 (2023). 10.1080/13682199.2023.2175426

[18] Cheng, C., Xu, T. & Wu, X.-J. Mufusion: A general unsupervised image fusion network based on memory unit. *Int. J. Inf. Fus.* **92**, 80–92. 10.1016/j.inffus.2022.11.010 (2023). 10.1016/j.inffus.2022.11.010

[19] Xu, H., Ma, J., Jiang, J., Guo, X. & Ling, H. U2fusion: A unified unsupervised image fusion network. *IEEE Trans. Pattern Anal. Mach. Intell.* **44**(1), 502–518. 10.1109/TPAMI.2020.3012548 (2022). 32750838 10.1109/TPAMI.2020.3012548

[20] Xu, H. & Ma, J. Emfusion: An unsupervised enhanced medical image fusion network. *Int. J. Inf. Fusion* **76**, 177–186. 10.1016/j.inffus.2021.06.001 (2021). 10.1016/j.inffus.2021.06.001

[21] Ma, J., Xu, H., Jiang, J., Mei, X. & Zhang, X.-P. Ddcgan: A dual-discriminator conditional generative adversarial network for multi-resolution image fusion. *IEEE Trans. Image Process.* **29**, 4980–4995. 10.1109/TIP.2020.2977573 (2020). 10.1109/TIP.2020.2977573 32167894

[22] Li, Q. *et al.* Coupled gan with relativistic discriminators for infrared and visible images fusion. *IEEE Sens. J.* **21**(6), 7458–7467. 10.1109/jsen.2019.2921803 (2021). 10.1109/jsen.2019.2921803

[23] Huang, J. *et al.* Mgmdcgan: Medical image fusion using multi-generator multi-discriminator conditional generative adversarial network. *IEEE Access Pract. Innov. Open Solut.* **8**, 55145–55157. 10.1109/access.2020.2982016 (2020). 10.1109/access.2020.2982016

[24] Lin, C., Mao, X., Qiu, C. & Zou, L. Dtcnet: Transformer-cnn distillation for super-resolution of remote sensing image. *IEEE J. Sel. Top. Appl. Earth Observ. Remote. Sens* **17**, 11117 (2024). 10.1109/JSTARS.2024.3409808

[25] Zhang, Y. *et al.* Ifcnn: A general image fusion framework based on convolutional neural network. *Int. J. Inf. Fusion* **54**, 99–118. 10.1016/j.inffus.2019.07.011 (2020). 10.1016/j.inffus.2019.07.011

[26] Xia, K.-J., Yin, H.-S. & Wang, J.-Q. A novel improved deep convolutional neural network model for medical image fusion. *Clust. Comput.* **22**(S1), 1515–1527. 10.1007/s10586-018-2026-1 (2019). 10.1007/s10586-018-2026-1

[27] Fu, J., Li, W., Du, J. & Huang, Y. A multiscale residual pyramid attention network for medical image fusion. *Biomed. Signal Process. Control* **66**, 102488. 10.1016/j.bspc.2021.102488 (2021). 10.1016/j.bspc.2021.102488

[28] Tang, W., He, F., Liu, Y. & Duan, Y. Matr: Multimodal medical image fusion via multiscale adaptive transformer. *IEEE Trans. Image Process.* **31**, 5134–5149. 10.1109/TIP.2022.3193288 (2022). 35901003 10.1109/TIP.2022.3193288

[29] Tang, L., Yuan, J. & Ma, J. Image fusion in the loop of high-level vision tasks: A semantic-aware real-time infrared and visible image fusion network. *Int. J. Inf. Fusion* **82**, 28–42. 10.1016/j.inffus.2021.12.004 (2022). 10.1016/j.inffus.2021.12.004

[30] Zhang, S. *et al.* Semantic-aware dehazing network with adaptive feature fusion. *IEEE Trans. Cybern.* **53**(1), 454–467. 10.1109/TCYB.2021.3124231 (2023). 34797770 10.1109/TCYB.2021.3124231

[31] Lee, Y., Jeon, J., Ko, Y., Jeon, B. & Jeon, M. Task-driven deep image enhancement network for autonomous driving in bad weather. In *2021 IEEE International Conference on Robotics and Automation (ICRA)* (IEEE, 2021).

[32] Ren, Y. *et al.* Multistage semantic-aware image inpainting with stacked generator networks. *Int. J. Intell. Syst.* **37**(2), 1599–1617. 10.1002/int.22687 (2022). 10.1002/int.22687

[33] Liu, D. *et al.* Connecting image denoising and high-level vision tasks via deep learning. *IEEE Trans. Image Process.* **29**, 3695–3706. 10.1109/TIP.2020.2964518 (2020). 10.1109/TIP.2020.2964518 31944972

[34] Haris, M., Shakhnarovich, G. & Ukita, N. *Task-Driven Super Resolution: Object Detection in Low-resolution Images* 387–395 (Springer, 2021).

[35] Tang, L., Deng, Y., Ma, Y., Huang, J. & Ma, J. Superfusion: A versatile image registration and fusion network with semantic awareness. *IEEE/CAA J. Autom. Sin.* **9**(12), 2121–2137. 10.1109/jas.2022.106082 (2022). 10.1109/jas.2022.106082

[36] Sun, Y., Cao, B., Zhu, P. & Hu, Q. Detfusion: A detection-driven infrared and visible image fusion network. In *Proc. 30th ACM International Conference on Multimedia* (ACM, 2022).

[37] Wang, P., Wang, M. & He, D. Multi-scale feature pyramid and multi-branch neural network for person re-identification. *Vis. Comput.*10.1007/s00371-022-02653-5 (2022).36185464 10.1007/s00371-022-02653-5

[38] Jia, Z. *et al.**MMCNN: A Multi-branch Multi-scale Convolutional Neural Network for Motor Imagery Classification* 736–751 (Springer, 2021).

[39] Chen, G., Dai, Y. & Zhang, J. C-net: Cascaded convolutional neural network with global guidance and refinement residuals for breast ultrasound images segmentation. *Comput. Methods Progr. Biomed.* **225**, 107086 (2022). 10.1016/j.cmpb.2022.107086 36044802

[40] Jiang, J. *et al.* Multibsp: Multi-branch and multi-scale perception object tracking framework based on Siamese cnn. *Neural Comput. Appl.*10.1007/s00521-022-07420-0 (2022).36467631 10.1007/s00521-022-07420-0

[41] Ghaderizadeh, S., Abbasi-Moghadam, D., Sharifi, A., Tariq, A. & Qin, S. Multiscale dual-branch residual spectral-spatial network with attention for hyperspectral image classification. *IEEE J. Sel. Top. Appl. Earth Observ. Remote Sens.* **15**, 5455–5467. 10.1109/jstars.2022.3188732 (2022). 10.1109/jstars.2022.3188732

[42] Li, W. *et al.* A multiscale double-branch residual attention network for anatomical–functional medical image fusion. *Comput. Biol. Med.* **141**(105005), 105005. 10.1016/j.compbiomed.2021.105005 (2022). 34763846 10.1016/j.compbiomed.2021.105005

[43] Chen, G., Dai, Y., Zhang, J., Yin, X. & Cui, L. Mbdsnet: Automatic segmentation of kidney ultrasound images using a multi-branch and deep supervision network. *Dig. Signal Process.* **130**, 103742 (2022). 10.1016/j.dsp.2022.103742

[44] Hu, J., Shen, L., Albanie, S., Sun, G. & Wu, E. Squeeze-and-excitation networks. *IEEE Trans. Pattern Anal. Mach. Intell.* **42**(8), 2011–2023. 10.1109/tpami.2019.2913372 (2020). 31034408 10.1109/tpami.2019.2913372

[45] Woo, S., Park, J., Lee, J.-Y. & Kweon, I. S. *CBAM: Convolutional Block Attention Module* 3–19 (Springer, 2018).

[46] Hou, Q., Zhou, D. & Feng, J. Coordinate attention for efficient mobile network design. In *2021 IEEE/CVF Conference on Computer Vision and Pattern Recognition (CVPR)* (IEEE, 2021).

[47] Thakur, R. K. & Maji, S. K. Agsdnet: Attention and gradient-based sar denoising network. *IEEE Geosci. Remote Sens. Lett.* **19**, 1–5. 10.1109/lgrs.2022.3166565 (2022). 10.1109/lgrs.2022.3166565

[48] Zhang, X., Zeng, H., Guo, S. & Zhang, L. Efficient long-range attention network for image super-resolution. In *Computer Vision—ECCV 2022* 649–667 (Springer, 2022).

[49] Cheng, B., Misra, I., Schwing, A. G., Kirillov, A. & Girdhar, R. Masked-attention mask transformer for universal image segmentation. In *2022 IEEE/CVF Conference on Computer Vision and Pattern Recognition (CVPR)* (IEEE, 2022).

[50] Lin, C., Qiu, C., Jiang, H. & Zou, L. A deep neural network based on prior driven and structural-preserving for sar image despeckling. *IEEE J. Select. Top. Appl. Earth Observ. Remote. Sens.* **1**, 1 (2023).

[51] Chen, G., Li, L., Dai, Y., Zhang, J. & Yap, M. H. Aau-net: An adaptive attention u-net for breast lesions segmentation in ultrasound images. *IEEE Trans. Med. Imaging* **42**, 1289 (2022). 10.1109/TMI.2022.3226268 36455083

[52] Wang, Z., Wu, Y., Wang, J., Xu, J. & Shao, W. Res2fusion: Infrared and visible image fusion based on dense res2net and double nonlocal attention models. *IEEE Trans. Instrum. Meas.* **71**, 1–12. 10.1109/tim.2021.3139654 (2022). 10.1109/tim.2021.3139654

[53] Ma, J. *et al.* Swinfusion: Cross-domain long-range learning for general image fusion via swin transformer. *IEEE/CAA J. Autom. Sin.* **9**(7), 1200–1217. 10.1109/jas.2022.105686 (2022). 10.1109/jas.2022.105686

[54] Liu, J., Shang, J., Liu, R. & Fan, X. Attention-guided global-local adversarial learning for detail-preserving multi-exposure image fusion. *IEEE Trans. Circuits Syst. Video Technol.* **32**(8), 5026–5040. 10.1109/tcsvt.2022.3144455 (2022). 10.1109/tcsvt.2022.3144455

[55] Hyun Cho, J., Mall, U., Bala, K. & Hariharan, B. Picie: Unsupervised semantic segmentation using invariance and equivariance in clustering. In *2021 IEEE/CVF Conference on Computer Vision and Pattern Recognition (CVPR)* (IEEE, 2021).

[56] Song, X., Wu, X.-J. & Li, H. *MSDNet for Medical Image Fusion* 278–288 (Springer, 2019).

[57] Zhang, H., Xu, H., Xiao, Y., Guo, X. & Ma, J. Rethinking the image fusion: A fast unified image fusion network based on proportional maintenance of gradient and intensity. *Proc. AAAI Conf. Artif. Intell.* **34**(07), 12797–12804. 10.1609/aaai.v34i07.6975 (2020). 10.1609/aaai.v34i07.6975

[58] Tang, W. & He, F. Fatfusion: A functional–anatomical transformer for medical image fusion. *Inf. Process. Manag.* **61**(4), 103687 (2024). 10.1016/j.ipm.2024.103687

[59] He, D., Li, W., Wang, G., Huang, Y. & Liu, S. Lrfnet: A real-time medical image fusion method guided by detail information. *Comput. Biol. Med.* **173**, 108381 (2024). 38569237 10.1016/j.compbiomed.2024.108381

[60] Xie, X. *et al.* Mrscfusion: Joint residual swin transformer and multiscale cnn for unsupervised multimodal medical image fusion. *IEEE Trans. Instrum. Meas.* **1**, 1 (2023).

